# A Concise and Divergent Approach to the Naturally Occurring Tetracyclic Clavine Alkaloids (+)-Lysergol, (+)-Lysergine, and (+)-Isolysergine

**DOI:** 10.1021/acs.joc.5c02229

**Published:** 2025-10-24

**Authors:** Alessio Regni, Francesca Bartoccini, Giovanni Piersanti

**Affiliations:** Department of Biomolecular Sciences, University of Urbino Carlo Bo, via Ca’ Le Suore 2, Urbino PU-61029, Italy

## Abstract

A concise and divergent asymmetric synthesis of the tetracyclic clavine alkaloids (+)-lysergol, (+) lysergine, and (+)-isolysergine has been accomplished using a novel strategy involving a chemoselective MeOH-mediated oxa-Michael addition and lactone–lactam rearrangement of appropriately functionalized spiro α-methylene-γ-butyrolactones, efficiently prepared from (*R*)-4-amino-Uhle’s ketone and bromomethyl acrylate. The straightforward downstream modifications complement and expand upon previous asymmetric total syntheses of these natural products.

## Graphical abstract

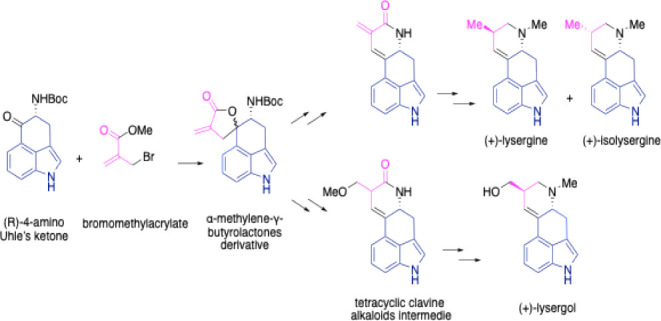

## Introduction

Ergot alkaloids (EAs) are a class of pharmacologically active indole compounds biosynthetically derived from l-tryptophan and dimethylallyl pyrophosphate, followed by cyclization.^1^ They are specialized fungal metabolites that are important in agriculture and serve as the basis for several pharmaceuticals.^2–6^ EAs are conveniently classified into two major structural groups: the medicinally valuable lysergic acid amide/peptide derivatives, and the biologically less explored clavines which are further subclassification based on their skeletons into tetracyclic, tricyclic and rearranged clavines (Figure 1a).^7,8^ Lysergic acid amide/peptide derivatives exhibit a wide range of biological activity due to their structural similarity to neurotransmitters and high binding affinity for various neurological receptors, including dopamine, adrenaline, and serotonin receptors.^9–15^ As a result, they have been extensively studied for over a century, leading to the development of drugs for treating migraine, depression, Parkinson’s disease, and postpartum hemorrhage.^16^ In contrast, relatively little is known about the biological activities of the clavine-derived EAs. Although these compounds are synthetic targets of interest not only due to their intriguing molecular architecture but also their proposed role as late-stage intermediates or derailment end products in the biosynthesis pathway of lysergic acid amide/peptide derivatives themselves, our understanding remains limited.^17^ This is likely due to the insufficient availability of material from direct extraction from natural sources, a process plagued by limitations such as dependence on environmental factors, fluctuating alkaloid content, and toxic crop-pathogenic ergot fungi.^18,19^ Moreover, the recent resurgence of interest in psychedelic compounds as potential therapeutics for neuropsychiatric disorders—such as addiction and anxiety—has further heightened attention on these molecules, which display intriguing biological profiles and promising medical applications.^20,21^

**Figure 1:**
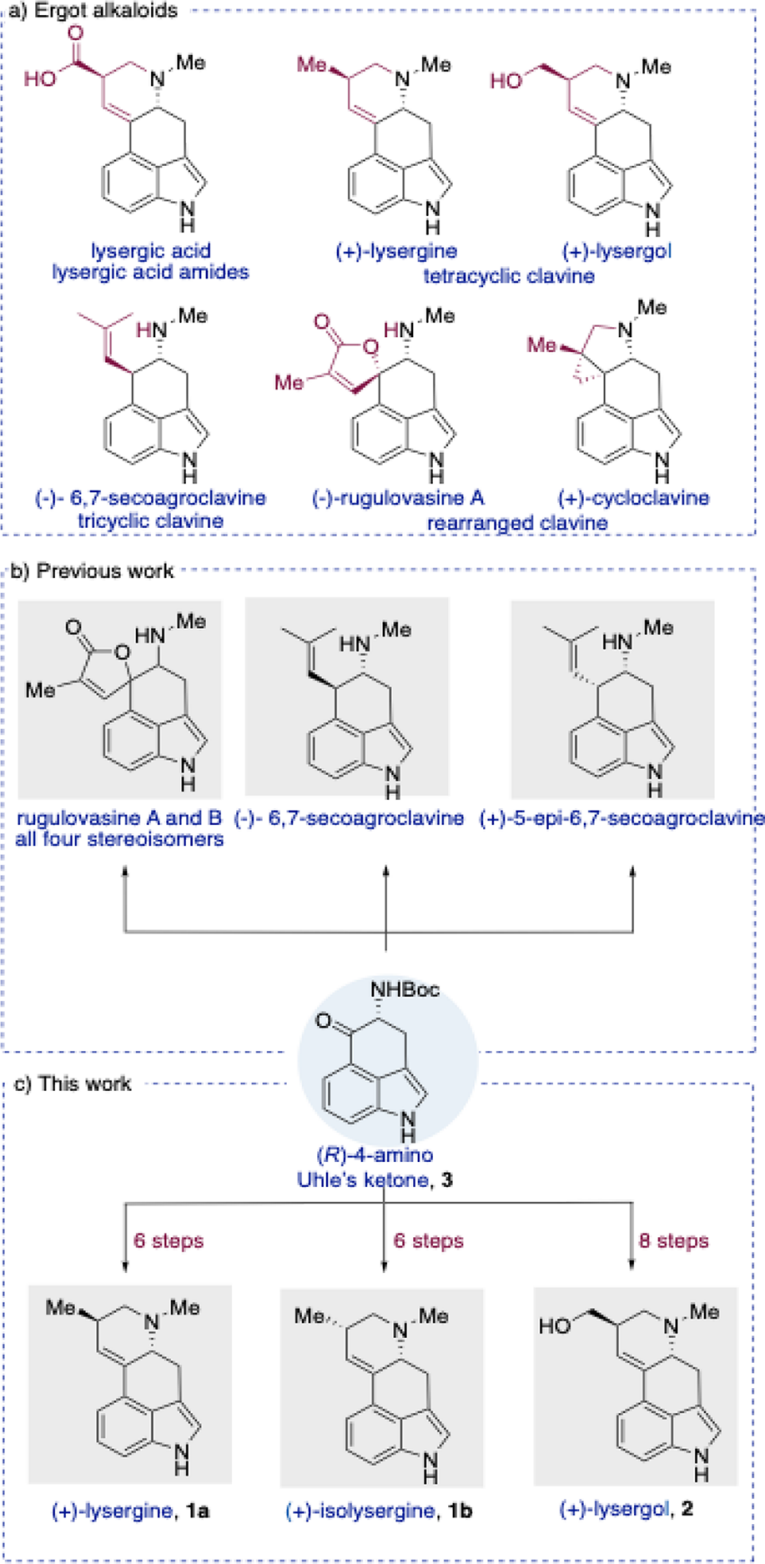
(a) Representative examples of naturally occurring ergot alkaloids. (b) Previous total syntheses based on (*R*)-4-amino Uhle’s ketone. (c) This work.

An illustrative example is the rearranged clavine (+)-cycloclavine, which exhibits enhanced selectivity for major central nervous system receptors compared to d-lysergic acid diethylamide (D-LSD),^22–24^ while maintaining strong affinity for dopaminergic and serotonergic receptors (5-HT1A and 5-HT2A). Another example is (+)-rugulovasine B, which demonstrates a substantially different 5-HT binding profile from D-LSD, yet retains high affinity and selectivity for 5-HT1A—an emerging and recently validated therapeutic target for neuropsychiatric disorders.^25,26^

Tetracyclic clavines are characterized by a complex tetracyclic framework that includes a tetrahydropyridine ring and a [cd]-fused/peri-annulated indole. These compounds contain multiple stereogenic centers with varying configurations, although the R-configuration at C-5 is invariant. The D-ring lacks a carboxyl group at C-8, which instead appears at the alcohol or alkane oxidation level, and may feature a double bond or not. This diversity makes tetracyclic clavines a compelling platform for developing new synthetic strategies and testing novel methodologies.^27^ For example, Wipf and co-workers have recently disclosed the shortest asymmetric synthesis of all four stereoisomers of lysergol and isolysergol, employing a transition metal-free hydrogen autotransfer alkylation protocol.^28^ In a complementary effort, Bisai and colleagues synthesized all stereoisomers of both natural and unnatural lysergine and isolysergine using an enantioselective organocatalytic α-aminoxylation strategy.^29^

In this context, our group has previously described novel reactivity paradigms enabling rapid access to rearranged and tricyclic clavines,^30,31^ including those derived from the chiral indole-based peri-annulated 4-amino-Uhle’s ketone scaffold (Figure 1b and 1c).^32^ Encouraged by these findings, we sought to expand the utility of enantiopure (*R*)-4-amino-Uhle’s ketone, accessible in four-step sequence from commercially available 4-boronate indole and d-serine-derived cyclic sulfamidate,^33^ in combination with a suitable four-carbon building block, toward a streamlined, concise, and divergent asymmetric synthesis of the ergoline tetracyclic core.

## Results and Discussion

Herein, we report the asymmetric syntheses of (+)-lysergine (**1a**), (+)-isolysergine (**1b**), and (+)-lysergol (**2**) (Figure 1c) via selective functional group manipulation, oxa-Michael addition, and lactone–lactam rearrangement of spiro α-methylene-γ-butyrolactones. All three EAs are readily accessible from (*R*)-4-amino-Uhle’s ketone and methyl 2-(bromomethyl)­acrylate.

The key intermediate, α-exomethylene-γ-butyrolactone **4**, serves as a branching point for divergence and possesses the necessary functionality for downstream transformations. Notably, it offers a low-energy pathway for D-ring construction via lactone-to-lactam rearrangement. Additionally, the lactone and electron-deficient alkene moieties in **4** provide ample opportunities for constructing valuable intermediates and functional group rearrangements.^34–39^

From a retrosynthetic perspective (Scheme 1), we envisioned introducing the *N*-methyl group found in all three target molecules, (+)-lysergine (**1a**), (+)-isolysergine (**1b**), and (+)-lysergol (**2**), via reductive amination of the dehydropiperidines **8a,b** and **(+)-13** with formaldehyde. These intermediates are obtained by reducing cyclic secondary lactams **(+)-7** and **(+)-12**, respectively. In the lysergine route, selective reduction of the exomethylene double bond in lactam **(+)-7** is targeted while preserving the endocyclic conjugated double bond within the D-ring. Regioselective elimination of the tertiary and benzylic alcohol **6**, present as a diastereomeric mixture, is expected to yield the thermodynamically preferred alkene. The D-ring is then constructed via a lactone ring-opening reaction followed by spontaneous, irreversible lactamization upon amine deprotection. Spiro α-methylene-γ-butyrolactone **4** is prepared through a Dreiding–Schmidt reaction between 2-bromomethyl acrylate ester and *N*-Boc-protected (*R*)-4-amino-Uhle’s ketone (**3**). The resulting compound features an electron-deficient alkene suitable for Michael-type additions with weak nucleophiles such as alcohols. In the lysergol route, a chemoselective oxa-Michael addition of MeOH to the exomethylene bond in compound **4** installs the necessary oxygen functionality for lysergol synthesis. From this point, the synthesis parallels that of lysergine, involving analogous lactone–lactam rearrangement, dehydration, amide reduction, reductive amination, and terminal alcohol deprotection.

**Scheme 1:**
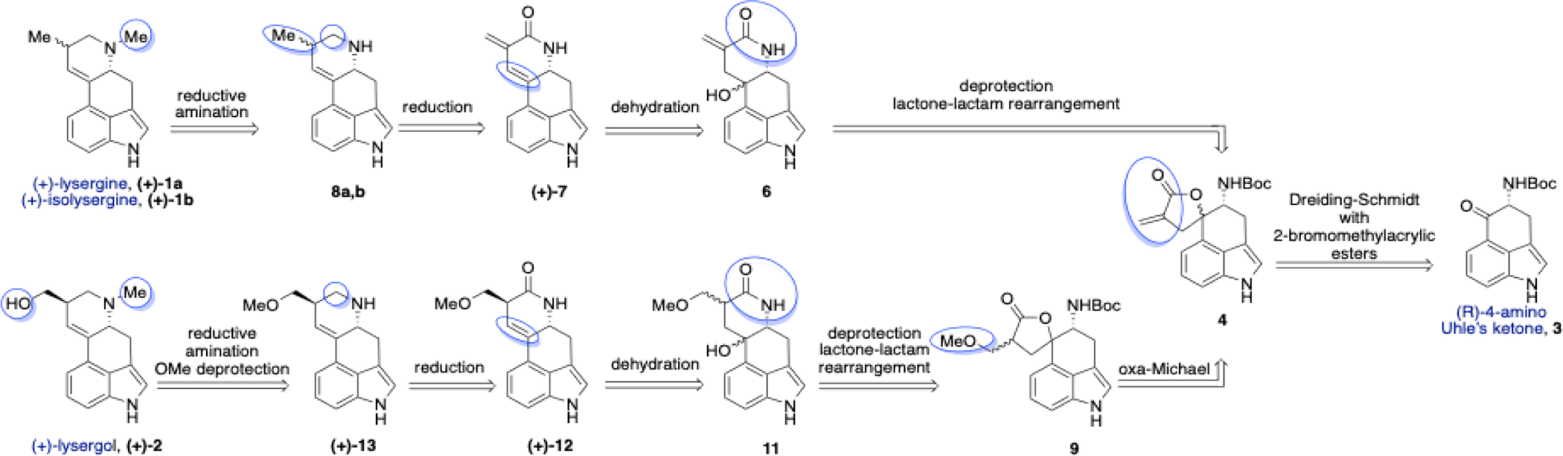
Retrosynthetic Analysis of (+)-Lysergine, (+)-Isolysergine, and (+)-Lysergol

For further simplification, we implemented a strategy based on a few one-pot operations.^40^ A key design feature was the one-pot formation of fully functionalized chiral tetracyclic fused lactam intermediates **(+)-7** and **(+)-12** through Boc deprotection, lactone–lactam rearrangement, and dehydration. Subsequent transformations—amide reduction and reductive amination—were also combined in one-pot procedures, minimizing isolation steps and maximizing efficiency.

We began our synthesis (Scheme 2) with the preparation of the α-methylene-γ-butyrolactone derivative **4** via the Dreiding–Schmidt reaction from (*R*)-4-amino-Uhle’s ketone (**3**) and 2-bromomethylacrylic ester, as previously reported.^25^ Although the diastereomers could be separated by flash chromatography on silica gel, we chose to proceed with the mixture, since the newly generated chiral center would be lost during later steps in favor of the *endo* double bond geometry. Early observations proved critical for informing the final synthetic route. Chemoselective ring-opening of the low-strain five-membered α-methylene γ-butyrolactone ring was achieved using excess KOH in a THF/H_2_O mixture at rt, yielding the corresponding carboxylate **15**. However, a dramatic difference in reactivity observed when the ring-opening conditions were slightly varied. When a large excess of MeONa (3 equiv), generated *in situ* from MeOH and NaH, was employed, only the MeOH addition product and the intact lactone **9a,a′,b,b′** were isolated in 95% yield.^41^ We speculated that a transient ring opening occurred,^42^ followed by a favorable 5-exo-trig cyclization to form the thermodynamically stable, saturated γ-butyrolactone **9**. Despite numerous attempts, we were unable to isolate or observe the ester intermediate. Furthermore, lactone **4a,b** was shown to be stable in MeOH without base, with neither ring-opening nor Michael addition observed after 24 h. Overall, these results demonstrate that direct chemoselective transformations of the α-methylene-γ-butyrolactone moiety in compound **4a,b** are indeed feasible. The reaction outcome—whether proceeding via ring-opening of the γ-lactone or MeOH addition via a Michael-type pathway—can be modulated by subtle changes in the reaction conditions. Nonetheless, under all conditions tested, the desired fourth-fused ring was not obtained from **4a,b**, neither via direct amidation (A) nor through an intramolecular aza-Michael addition involving the neighboring nitrogen lone pair (B).

**Scheme 2:**
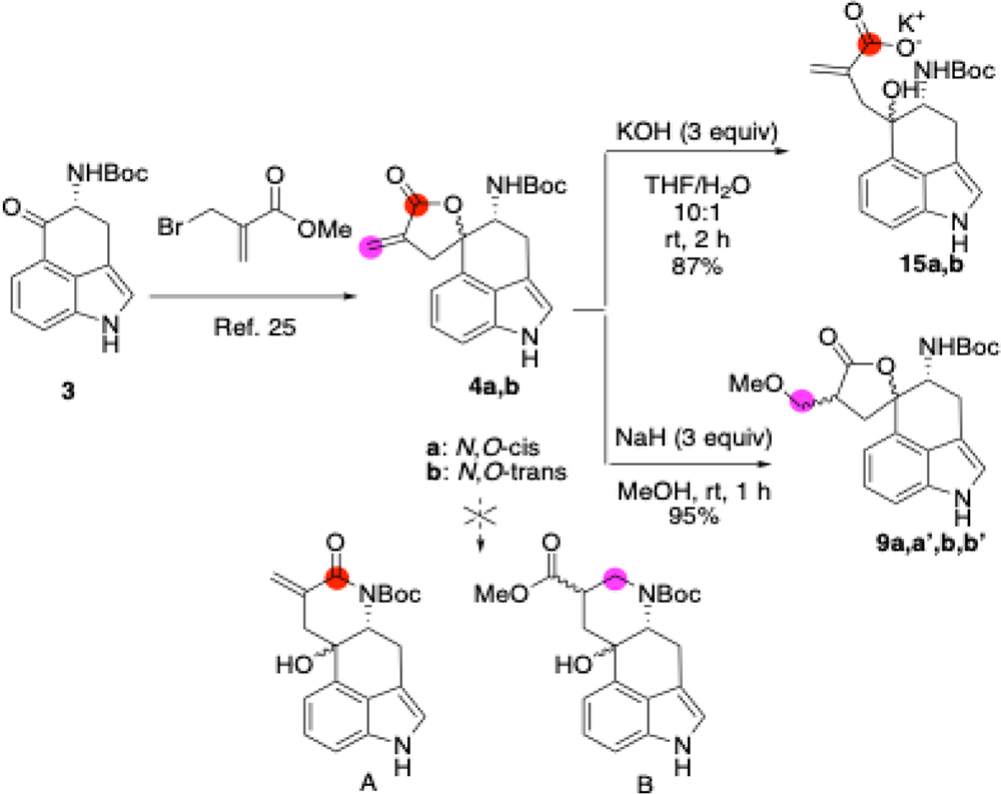
Initial Efforts to Construct the Fourth Fused Ring of the Ergoline Core

Given the well-established high nucleophilicity of amino groups relative to the poor nucleophilicity of carbamates, as well as numerous examples of amines facilitating low-energy acyl transfer pathways and the thermodynamic preference for amide formation, we explored a more promising strategy centered on a lactone–lactam rearrangement.^43,44^ This approach was designed to generate the desired unsaturated six-membered lactam via the free amine intermediate.

Boc deprotection of compounds **4a,b** and **9a,a′,b,b′** was carried out without chromatographic purification. Treatment with 4 M HCl in dioxane yielded the corresponding amine hydrochloride salts (Scheme 3).

**Scheme 3:**
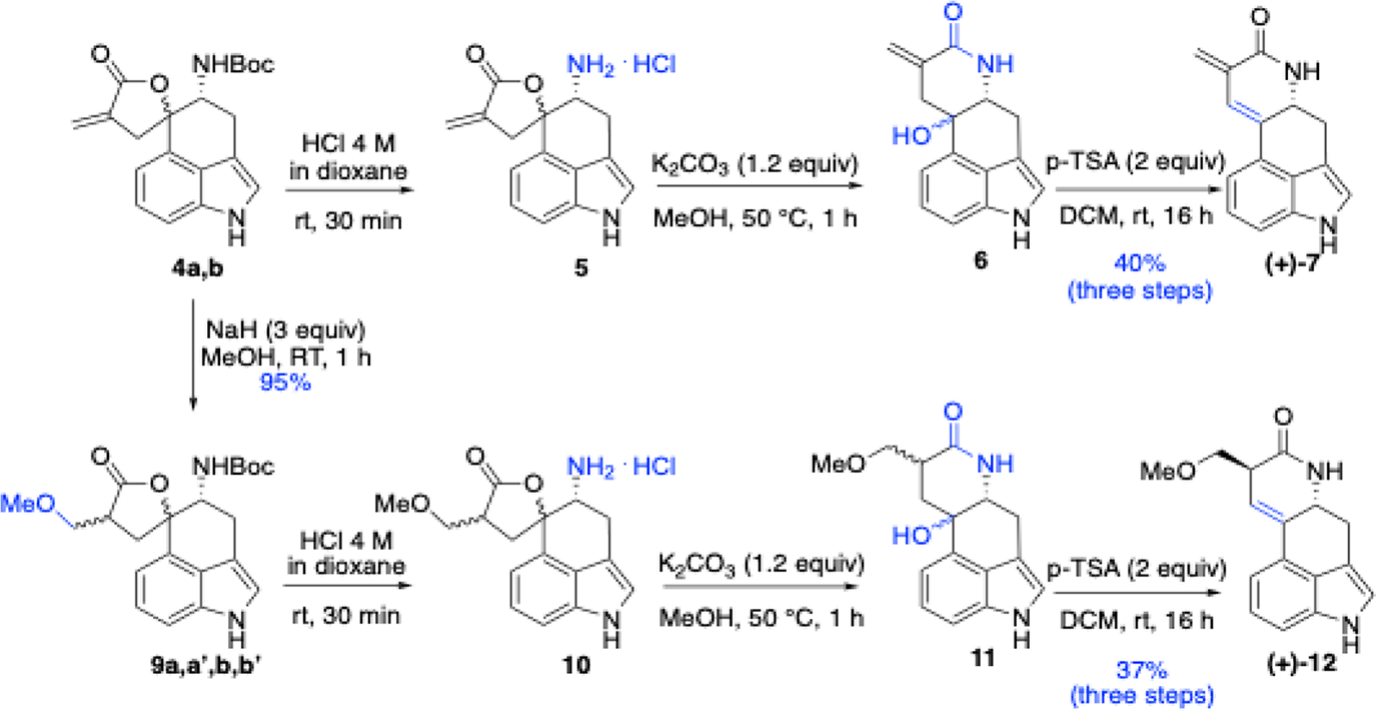
Synthesis of **(+)-7** and **(+)-12** by Lactone–Lactam Rearrangement and Dehydration

We then investigated whether the α-methylene spirocyclic butyrolactone subunit could be converted into the fully fused tetracyclic core of (+)-lysergine (**1a**) and (+)-lysergol (**2**) through a lactone–lactam rearrangement (cf. Scheme 1). Inspired by a previous report by Rebek,^45^ who induced such an acyl transfer in a related dihydroindole system containing a lactone with an endocyclic double bond, toward the synthesis of (±)-setoclavine, we found that treating the amine salts of α-exomethylene spirocyclic butyrolactone derivatives **5** and **10** with 1.2 equiv of K_2_CO_3_ in MeOH at 50 °C yielded a new isomeric intermediate. Subsequent treatment with *p*-toluenesulfonic acid in CH_2_Cl_2_ furnished the unusual diene (**+)-7** and the ether **(+)-12** in 37% and 40% overall yield (over three steps), respectively.^46^ We propose that formation of the fourth fused ring proceeds via initial methanolysis of the lactone to an ester, followed by a rapid and favorable 6-exo-trig cyclization to form the lactam.^47^ Remarkably, this transformation represents an advantageous atom-economical pathway that bypasses the hydrolysis and activation steps typically required for conventional amide bond formation.^48^ This rearrangement also liberates a hydroxyl group that can subsequently undergo dehydration. It is noteworthy that compound **(+)-12** was obtained as a single stereoisomer, which is consistent with the known tendency of amido carbonyl systems—such as peptides—to epimerize toward the thermodynamically favored stereoisomer under the reaction conditions.

Both syntheses proceeded smoothly, though selective reduction of lactams **(+)-7** and **(+)-12** to their corresponding amines required careful condition optimization (Schemes 4–5). Initial attempts employing common reducing agents—LiAlH_4_ (with or without AlCl_3_), DIBAL-H, NaBH_4_ and BH_3_—under various solvents and temperatures led either to degradation of the starting materials or incomplete reduction of the exocyclic conjugated double bonds. We successfully applied a method developed by Geng and co-workers,^49^ which uses only two reagents: triflic anhydride (Tf_2_O) and NaBH_4_. This approach involves chemoselective activation of the secondary lactam using Tf_2_O in THF at rt for 30 min, followed by reduction with excess NaBH_4_ under the same conditions. The process afforded secondary free amine **8a,b** as a mixture of diastereomers—suitable for subsequent conversion to both lysergine and isolysergine. The crude amine mixture was directly subjected to reductive amination using formalin, acetic acid, and NaCNBH_3_ in MeOH, producing a separable mixture of (+)-lysergine (**1a**) and (+)-isolysergine (**1b**). We optimized these two steps into a one-pot protocol, yielding the final products in 50% overall yield. Characterization data for both alkaloids were consistent with literature values. The ^1^H NMR spectra perfectly matched reported data for (+)-lysergine^44^ and (+)-isolysergine.^50^ Optical rotation measurements were likewise in agreement with the reported values for (+)-lysergine (observed [α]_D_^20^ = +61.4 (c = 0.045, pyridine); Lit.^51^ [α]_D_^20^ = +70 (c = 0.2, pyridine); (+) Isolysergine, (observed [α]_D_^20^ = +196 (c = 0.055, pyridine); Lit.^52^ [α]_D_^20^ = +200 (c = 0.5, pyridine).

**Scheme 4:**
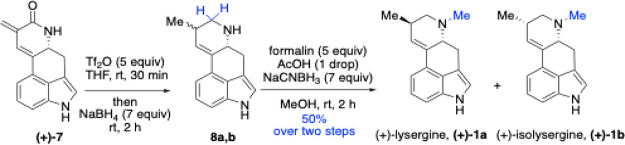
Synthesis of (+)-Lysergine (**1a**) and (+)-Isolysergine (**1b**)

**Scheme 5:**
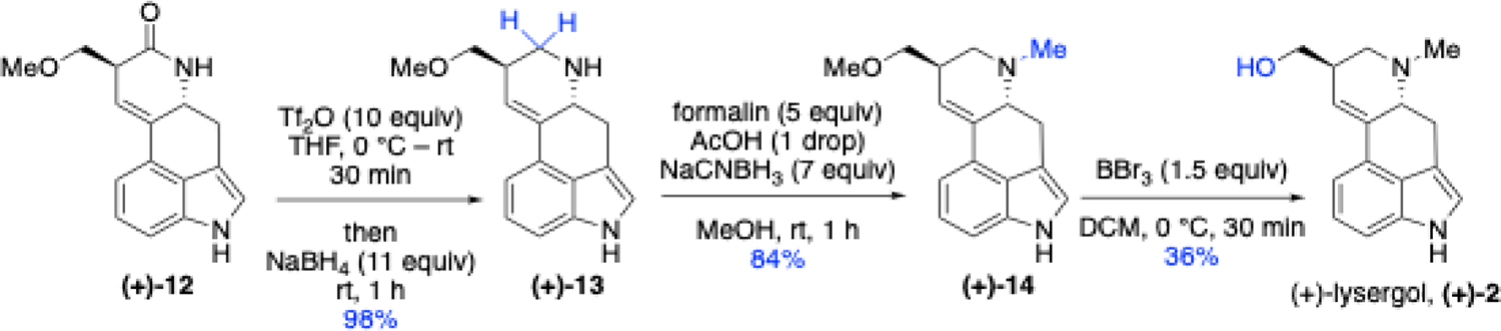
Synthesis of (+)-Lysergol (**(+)-2**)

The same reaction sequence was applied for the synthesis of (+)-lysergol (**2**) (Scheme 5). Reduction of amide **(+)-12** using Tf_2_O and NaBH_4_, followed by reductive amination, furnished the tertiary amine **(+)-14**. The final step involved removal of the methoxy protecting group to yield the primary alcohol. Initially, we attempted the deprotection conditions reported by Garg and co-workers in their total synthesis of dragmacidin D,^53^ which employed trimethylsilyl iodide in MeCN. However, these conditions resulted in degradation of the starting material. We then successfully employed boron tribromide in CH_2_Cl_2_, which afforded the target compound (+)-lysergol (**2**) as a single enantiomer in 36% yield. All characterization data were consistent with the literature. The ^1^H NMR spectrum of (+)-lysergol matched that reported previously.^28^ Furthermore, the optical rotation was also consistent with the reported values: (+)-lysergol (observed [α]_D_^25^ = +62.5 (c = 0.12, MeOH); Lit.^28^ [α]_D_^25^ = +66.8 (c = 0.32, MeOH).

## Conclusions

In conclusion, we completed concise, divergent, and asymmetric syntheses of (+)-lysergine (**1a**), (+)-isolysergine (**1b**), and (+)-lysergol (**2**). The key innovation of this strategy lies in the strategic combination of an indole-containing peri-annulated scaffold, (*R*)-4-amino-Uhle’s ketone (**3**), with 2-bromomethyl acrylate to construct the common intermediate spiro α-methylene-γ-butyrolactone **4**, which bears the core functionality and skeletal features necessary for the synthesis of these alkaloids. In addition, this route leverages a methoxide-mediated lactone–lactam rearrangement to construct the ergoline tetracyclic system, circumventing the need for conventional coupling reagents. This study also underscores the reactivity of α-methylene-γ-butyrolactones, particularly the exomethylene moiety, which undergoes a chemoselective intermolecular oxa-Michael addition with MeOH. The synthetic sequences proceed in decent yield (LLS 10 steps with 3.3% yield for (+)-lysergine (**1a**) and (+)-isolysergine (**1b**), and LLS 12 steps with 3.4% yield for (+)-lysergol (**2**)) with minimal purification. These syntheses not only validate the utility of (*R*)-4-amino-Uhle’s ketone as a versatile chiral building block for complex natural products but also establish a broadly applicable platform for future alkaloid syntheses. Further applications of this methodology are currently under investigation and will be reported in due course.

## Supplementary Material



## Data Availability

The data underlying this study are available in the published article and its Supporting Information

## Notes

General information, experimental details, characterization of compounds, tables for comparison, ^1^H NMR data for synthetic compounds with that of natural products, and copies of ^1^H and ^13^C NMR spectra (PDF)

## †

Department of Chemistry, Princeton University, Princeton, NJ 08544, USA (A.R.)
