# Supplementary material for: A Concise and Divergent Approach to the Naturally Occurring Tetracyclic Clavine Alkaloids (+)-Lysergol, (+)-Lysergine, and (+)-Isolysergine
Source: J Org Chem. 2025 Oct 24;90(44):15861–6. doi: 10.1021/acs.joc.5c02229 (PMC12604026; doi:10.1021/acs.joc.5c02229)
Supplement: Supplementary file 1 [file jo5c02229_si_001.pdf]

Supporting Information

A Concise and Divergent Approach to the Naturally Occurring Tetracyclic Clavine Alkaloids (+)-Lysergol, (+)-Lysergine, and (+)-Isolysergine

Alessio Regni, Francesca Bartoccini and Giovanni Piersanti*

University of Urbino Carlo Bo, Department of Biomolecular Sciences, via Ca' Le Suore 2, PU-61029 Urbino, (Italy).

E-mail: giovanni.piersanti@uniurb.it

Homepage: https://sites.google.com/uniurb.it/giovannipiersanti

Table of Contents

1. General Methods S2
2. Starting Materials S2
3. Synthesis and characterization data of compound **4a,b** S3
4. Synthesis and characterization data of compound **15a,b** S3
5. GP1: General procedure for oxa-Michael reaction S4
6. Synthesis and characterization data of compounds **9a,a'** and **9b,b'** S4
7. GP2: General procedure for deprotection, lactone–lactam rearrangement and dehydration. S5
8. Synthesis and characterization data of compound **(+)-7** S5
9. Synthesis and characterization data of compound **(+)-12** S6
10. Synthesis and characterization data of (+)-lysergine **((+)-1a)** and (+)-isolysergine **((+)-1b)** S6
11. Synthesis and characterization data of compounds **(+)-13** S7
12. Synthesis and characterization data of compounds **(+)-14** S8
13. Synthesis and characterization data of (+)-lysergol **(+)-2** S8
14. Table S1. Comparison of our ^{1}H NMR data of compound **(+)-1a** with literature data S10
15. Table S2. Comparison of our ^{1}H NMR data of compound **(+)-1b** with literature data S11
16. Table S3. Comparison of our ^{1}H NMR data of compound **(+)-2** with literature data S12
17. References S13
18. Copies of ^{1}H NMR and ^{13}C{1H} NMR spectra S14

GENERAL METHODS

All reactions were run in air unless otherwise noted. Without special instructions, the heating reactions use an oil bath. Column chromatography purifications were performed in flash chromatography conditions using 230-400 Mesh silica gel or 70- 230 Mesh silica gel. Analytical thin layer chromatography (TLC) was carried out on silica gel plates (Silica Gel 60 F254) that were visualized by exposure to ultraviolet light and an aqueous solution of *p*-anisaldehyde. ^{1}H NMR, ^{13}C{1H} NMR and ^{11}B NMR spectra were recorded on 400 MHz spectrometer, using $CDCl_3$, DMSO-*d6,* MeOH-d_4 and D_2O, as solvents. Chemical shifts (δ scale) are reported in parts per million (ppm) relative to the central peak of the solvent. Coupling constants (*J* values) are given in hertz (Hz). Melting points were determined on the capillary melting point apparatus and were uncorrected. Optical rotation analysis was performed with a polarimeter using a sodium lamp (λ 589 nm, D-line); $[\alpha]_D^{20}$ values are reported in 10^{-1} deg cm^2 g^{-1}; concentration (c) is in g for 100 mL. HRMS analysis was performed using Orbitrap Exploris Mass Spectrometers.

STARTING MATERIALS

tert-Butyl (*R*)-(5-oxo-1,3,4,5-tetrahydrobenzo[*cd*]indol-4-yl)carbamate (**3**) is synthetized as reported in the literature.[1]

***tert*-Butyl ((4*R*,5*S*)-4'-methylene-5'-oxo-3,4,4',5'-tetrahydro-1*H*,3'*H*-spiro[benzo[*cd*]indole-5,2'-furan]-4-yl)carbamate (4a) and *tert*-butyl ((4*R*,5*R*)-4'-methylene-5'-oxo-3,4,4',5'-tetrahydro-1*H*,3'*H*-spiro[benzo[*cd*]indole-5,2'-furan]-4-yl)carbamate (4b).**

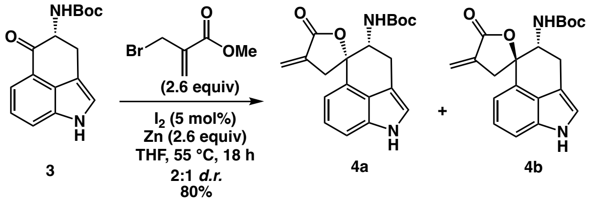

Compound **3** (1 g, 3.5 mmol) was dissolved in THF (50 mL) and cooled to 0 °C with stirring under N_2. To this solution was added I_2 (44 mg, 0.175 mmol), activated Zn powder (595 mg, 9.1 mmol) and ethyl α-(bromomethyl)acrylate (0.88 ml, 7.35 mmol). The mixture was stirred at 55 °C for 18 h. Then saturated aqueous NH_4Cl was added, and the aqueous phase was extracted with DCM (3 x 50 mL). The combined organic phases were washed with brine, dried over Na_2SO_4, filtered, and the solvent evaporated under reduced pressure. The residue obtained was purification by flash-chromatography (cyclohexane/EtOAc 8:2) to afford **4a,b** as a mixture of separable diastereomers (diastereomeric ratio *cis:trans* 2:1). **4a** (656 mg, 53%) and **4b** (334 mg, 27%) were isolated as yellow solids. **4a**: ^{1}H NMR: (400 MHz, $CDCl_3$) δ 8.23 (br s, 1H), 7.33 (d, *J* = 8.0 Hz, 1H), 7.20 (d, *J* = 7.5 Hz, 1H), 7.08 (d, *J* = 7.5 Hz, 1H), 6.96 (s,1H), 6.33 (s, 1H), 5.75 (s, 1H), 4.80 (br d, 1H), 4.35 – 4.29 (m, 1H), 3.44 – 3.34 (m, 2H), 3.16 (dd, *J* = 15.5, 5.0 Hz, 1H), 3.06 (dd, *J* = 15.5, 9.5 Hz, 1H), 1.45 (s, 9H). **4b**: ^{1}H NMR: (400 MHz, $CDCl_3$) δ 8.55 (br s, 1H), 7.31 (d, *J* = 8.0 Hz, 1H), 7.17 (t, *J* = 7.5 Hz, 1H), 7.07 (d, *J* = 7.5 Hz, 1H), 6.97 (s,1H), 6.36 (s, 1H), 5.81 (s, 1 H), 4.47 (s, 2H), 3.55 (br s, 1H), 3-43 – 3.35 (m, 2H), 2.94 (d, *J* = 15.0 Hz, 1H), 1.39 (s, 9H); The chemical–physical data are in according to the compounds reported in the literature.[2]

Potassium 2-(((4*R*)-4-((*tert*-butoxycarbonyl)amino)-5-hydroxy-1,3,4,5-tetrahydrobenzo[*cd*]indol-5-yl)methyl)acrylate (15a,b)

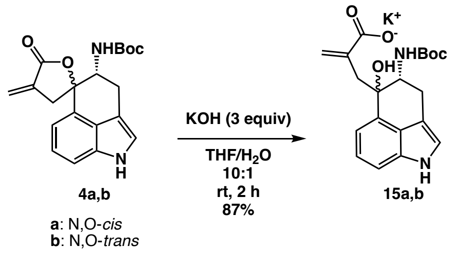

To a solution of **4a,b** (10 mg, 0.028 mmol) in THF dry (0.2 mL) was added a solution of KOH (6.9 mg, 0.123 mmol) in H_2O (15 μl). The reaction mixture was stirred at room temperature for 2 h. The volatiles were evaporated under reduced pressure to obtained **15a,b** (10 mg, 87%) as a white amorphous solid ^{1}H NMR (600 MHz, D_2O) δ (Only the major diastereomer was reported) 7.38 (d, *J* = 7.5, Hz, 1H), 7.24 (t, *J* = 7.5 Hz, 1H), 7.13 – 7.10 (m, 2H), 5.86 (s, 1H), 5.09 (s, 1H), 4.21 (s,

1H), 3.34 – 3.32 (m, 1H), 2.96 – 2.95 (m, 1H), 2.83 (s, 2H), 1.34 (s, 9H). $^{13}C\{1H\}$ NMR (150 MHz, D_2O) δ 182.8, 160.6, 141.0, 133.6, 125.2, 122.5, 122.4, 121.0, 115.1, 110.4, 110.1, 107.2, 80.7, 74.5, 55.3, 27.7, 27.6, 26.4. HRMS (ESI-TOF) m/z: $[M - H]^-$ calcd for $C_{20}H_{23}N_2O_5$, 371.1612; found, 371.1621.

GP1: General procedure for oxa-Michael addition.

To a solution of the appropriate compound **4a** or **4b** (1 equiv) in MeOH (0.2 M) was added NaH (3 equiv) previous remotion of the mineral oil by treating the NaH 60% in mineral oil with hexanes and decantated it three times. The mixture was stirred at room temperature for 1 h. The mixture was diluted with NH_4Cl saturated solution, and the solution was extracted with DCM three times. The combined organic phases were washed with brine and dried over Na_2SO_4, filtered, and the solvent evaporated under reduced pressure. The residue obtained was purified by flash chromatography.

***tert*-Butyl((4*R*,4'*S*,5*S*)-4'-(methoxymethyl)-5'-oxo-3,4,4',5'-tetrahydro-1*H*,3'*H*-spiro[benzo[*cd*] indole-5,2'-furan]-4-yl)carbamate (9a) and *tert*-butyl ((4*R*,4'*R*,5*S*)-4'-(methoxymethyl)-5'-oxo-3,4,4',5'-tetrahydro-1*H*,3'*H*-spiro[benzo[*cd*]indole-5,2'-furan]-4-yl)carbamate (9a').**

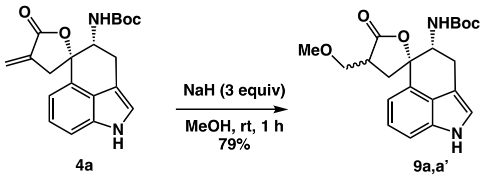

The title compounds were synthesized according to the general procedure GP1 using **4a** as starting material (30 mg, 0.085 mmol). The residue was purified by flash chromatography (cyclohexane/EtOAc 8:2) to obtain an inseparable mix of diastereoisomers **9a,a'** in 1:2.5 ratio (26 mg, 79%) as white amorphous solid. ^1H NMR (400 MHz, DMSO-d_6) δ 10.89 (s, 3.5H), 7.35-7.32 (m, 3H), 7.13-7.07 (m, 11H), 4.11-4.04 (m, 1H), 4.01-3.91 (m, 2.5H), 3.70 (dd, J = 4.0, 8.0 Hz, 1H), 3.67-3.58 (m, 6.5H), 3.54-3.44 (m, 5H), 3.30 (s, 10.5H), 3.06-2.80 (m, 9.5H), 2.72-2.60 (m, 3.5H), 2.59-2.52 (m, 2.5H) 1.41 (s, 31.5H). $^{13}C\{1H\}$ NMR (100 MHz, DMSO-d_6): δ 176.20, 176.17, 156.1, 155.6, 133.8, 130.0, 129.1, 126.3, 125. 7, 122.1, 122.0, 119.9, 119.8, 114.1, 113.8, 111.7, 111.6, 108.60, 108.56, 84.5, 78.4, 78.1, 71.0, 70.7, 58.6, 58.4, 55.3, 53.8, 42.0, 42.0, 28.23, 28.19, 25.6, 25.1. HRMS (ESI-TOF) m/z: $[M + Na]^+$ calcd for $C_{21}H_{26}N_2NaO_5$, 409.1734; found, 409.1736.

***tert*-butyl ((4*R*,4'*S*,5*S*)-4'-(methoxymethyl)-5'-oxo-3,4,4',5'-tetrahydro-1*H*,3'*H*-spiro[benzo[*cd*]indole-5,2'-furan]-4-yl)carbamate (9b) and *tert*-butyl ((4*R*,4'*R*,5*S*)-4'-(methoxymethyl)-5'-oxo-3,4,4',5'-tetrahydro-1*H*,3'*H*-spiro[benzo[*cd*]indole-5,2'-furan]-4-yl)carbamate (9b')**

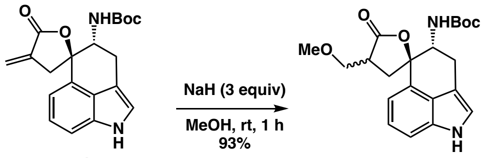

4b 9b,b'

The title compound was synthesized according to the general procedure GP1 using **4b** as starting material (63 mg, 0.18 mmol). The residue was purified by flash chromatography (cyclohexane/EtOAc 8:2) to obtain **9b,b'** (65 mg, 93%) a separable mix of diastereoisomers as white amorphous solid.

Apolar spot: ^{1}H NMR (400 MHz, DMSO-*d₆*) δ 10.87 (d, *J* = 2.0 Hz, 1H), 7.25 (d, *J* = 8.0 Hz, 1H), 7.11 – 7.07 (m, 2H), 7.06 (d, *J* = 2.0 Hz, 1H), 6.86 (d, *J* = 7.0 Hz, 1H), 4.15 – 4.07 (m, 1H), 3.64 (q, *J* = 5 Hz, 1H), 3.50 (dd, *J* = 6.0, 3.0 Hz, 1H), 3.27 (s, 3H), 3.12–3.06 (m, 1H), 2.97 – 2.95 (m, 2H), 2.77 (dd, *J* = 10.5, 3.0 Hz, 1H), 2.02 – 1.96 (m, 1H), 1.37 (s, 9H); ^{13}C{1H} NMR (100 MHz, DMSO-d_6): δ 176.7 155.2, 133.5, 125.1, 122.3, 119.6, 113.7, 112.0, 110.3, 107.8, 85.3, 78.1, 70.9, 58.5, 54.1, 42.0, 33.3, 28.2, 26.0; $[\alpha]_D^{20}$ = -45,4 (c = 0.15, $CHCl_3$). HRMS (ESI-TOF) m/z: $[M + Na]^+$ calcd for $C_{21}H_{26}N_2NaO_5$, 409.1734; found, 409.1736.

Polar spot: ^{1}H NMR (400 MHz, DMSO-d_6) δ 10.89 (br s, 1H), 7.28 (d, *J* = 8.0 Hz, 1H), 7.11-7.07 (m, 3H), 6.84 (m, 1H), 4.19 – 4.13 (m, 1H), 3.69 – 3.57 (m, 2H), 3.38 – 3.25 (m, 5H), 3.02 (dd, *J* = 16.0, 5.0 Hz, 1H), 2.92 (dd, *J* = 16.0, 10.0 Hz, 1H), 2.59 (t, *J* = 12.0 Hz, 1H), 1.37 (s, 9H). ^{13}C{1H} NMR (100 MHz, DMSO-d_6): δ 176.5, 155.2, 133.8, 124.9, 122.1, 122.0, 120.0, 119.9, 110.8, 108.0, 84.4, 78.0, 70.5, 58.3, 53.8, 31.9, 29.0, 28.2, 25.9; $[\alpha]_D^{20}$ = -57,3 (c = 0.13, $CHCl_3$). HRMS (ESI-TOF) m/z: $[M + Na]^+$ calcd for $C_{21}H_{26}N_2NaO_5$, 409.1734; found, 409.1736.

GP2: General procedure for deprotection, lactone–lactam rearrangement and dehydration. To a solution of the appropriate compound **4a,b** or **9a,a',b,b'** (1 equiv) was added 4 M HCl in dioxane (0.2M). The mixture was stirred at room temperature for 30 minutes. Completion of the reaction was confirmed by TLC (Cyclohexane/EtOAc 7:3). The volatiles were removed under reduced pressure, and the crude residue was dissolved in MeOH (0.2 M). K_2CO_3 (1.2 equiv) was added, and the reaction was stirred at 50 °C for 1 hour. After the reaction was completed (checking TLC DCM:MeOH 95:5) acetic acid (0.5 mL) was added, and the solvent was removed under reduced pressure. The crude was dissolved in DCM and evaporated three times. The resulting residue was dissolved in DCM (0.13 M), and *p*-TSA (2 equiv) was added. The reaction was stirred at room temperature for 16 hours. The solvent was removed under reduced pressure, and the crude product was purified by flash chromatography.

(*R*)-9-Methylene-4,6a,7,9-tetrahydroindolo[4,3-*fg*]quinolin-8(6*H*)-one ((+)-7)

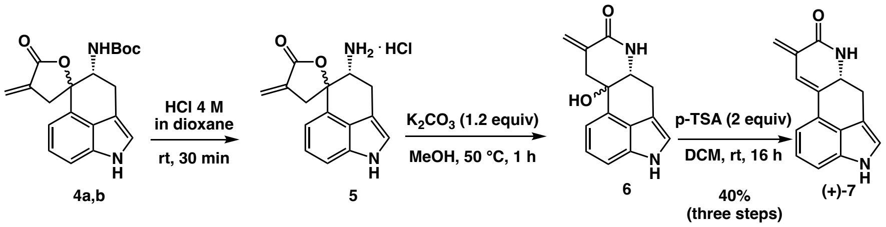

The title compound was synthesized according to the general procedure GP2 using **4a,b** (100 mg, 0.28 mmol) as starting material. The residue was purified by flash-chromatography (DCM/MeOH 98:2) to afford product **(+)-7** (26 mg, 40%) as yellowish amorphous solid. ^{1}H NMR (400 MHz, MeOH-d_4) δ 7.28 – 7.23 (m, 2H), 7.16 (t, J = 8.0 Hz, 1H), 7.08 (s, 1H), 7.01 (s,1H), 6.15 (s, 1H), 5.54 (s, 1H), 4.65 – 4.60 (m, 1H), 3.41 (dd, J = 14.5, 6.0 Hz, 1H), 3.86 (dd, J = 14.5, 2.5 Hz, 1H). ^{13}C{1H} NMR (100 MHz, MeOH-d_4): δ 162.8, 132.9, 132.6, 132.4, 125.2, 124.4, 120.9, 117.7, 117.6, 117.5, 110.1, 109.1, 106.7, 53.1, 29.1; $[\alpha]_D^{20}$ = +22,6 (c = 0.075, MeOH). HRMS (ESI-TOF) m/z: $[M + Na]^+$ calcd for $C_{15}H_{12}N_2NaO$, 259.0842; found, 259.0839.

(6a*R*,9*S*)-9-(Methoxymethyl)-4,6a,7,9-tetrahydroindolo[4,3-*fg*]quinolin-8(6*H*)-one ((+)-12)

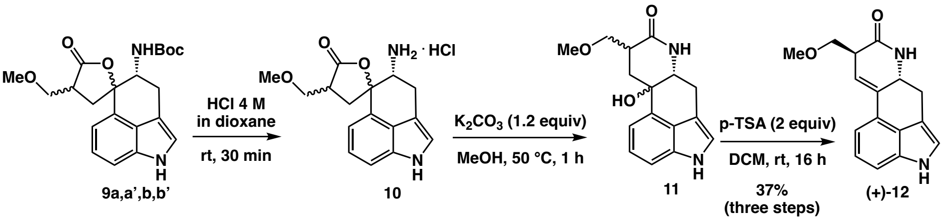

The title compound was synthesized according to the general procedure GP2 using **9a,a',b,b'** (80 mg, 0.23 mmol) as starting material. The residue was purified by flash-chromatography (DCM/MeOH 98:2) to afford product **(+)-12** (23 mg, 37%) off-white amorphous solid. ^{1}H NMR (400 MHz, MeOH-d_4) δ 7.24 (d, J = 7.5 Hz, 1H), 7.19 – 7.11 (m, 2H), 6.97 (s, 1H), 6.34 (br s, 1H), 4.48 – 4.44 (m, 1H), 3.88 (dd, J = 9.0, 5.0 Hz, 1H), 3.70 (dd, J = 9.0, 3.5 Hz, 1H), 3.39 (s, 3H), 3.27 – 3.18 (m, 1H), 2.81 – 2.74 (m, 1H). ^{13}C{1H} NMR (100 MHz, MeOH-d_4): δ172.8, 135.8, 134.0, 127.8, 127.6, 123.7, 120.1, 118.6, 112.6, 111.2, 109.8, 74.9, 59.5, 54.9, 44.0, 32.1; $[\alpha]_D^{20}$ = +5 (c = 0.2, MeOH). HRMS (ESI-TOF) m/z: $[M + Na]^+$ calcd for $C_{16}H_{16}N_2NaO_2$, 291.1104; found, 291.1098.

Lysergine ((+)-1a) and isolysergine ((+)-1b)

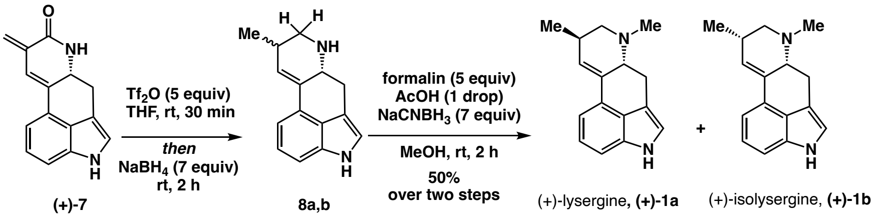

To a solution of compound **(+)-7** (10 mg, 0.042 mmol) in THF (0.78 mL, 0.05 M), at 0 °C under N_2, Tf_2O (0.21 mmol, 22 µL) was added dropwise. The reaction mixture was stirred at 0 °C for 30 minutes, and $NaBH_4$ (0.294 mmol, 11 mg) was added portion-wise. The reaction was then allowed to warm to room temperature and stirred for 2 hours. The reaction was quenched with a saturated solution of Na_2CO_3, and the mixture was extracted four times with a solution of DCM:MeOH (9:1). The combined organic phases were washed with brine, dried over Na_2SO_4, filtered, and the solvent evaporated under reduced pressure. The residue obtained was dissolved in MeOH (0.78 mL, 0.05 M) and formalin (0.05 mL, 0.21 mmol), AcOH (1 drop), and $NaCNBH_3$ (18 mg, 0.294 mmol) were added. The reaction mixture was stirred at room temperature for 2 hours. The reaction was diluted with a saturated solution of Na_2CO_3 and extracted four times with a DCM:MeOH (9:1). The combined organic phases were washed with brine, dried over Na_2SO_4, filtered, and the solvent evaporated under reduced pressure. The mixture of diastereoisomers **(+)-1a** (2.5 mg, 25%) and **(+)-1b** (2.5 mg, 25%) was isolated by preparative TLC (DCM/MeOH 95:5) as off-white amorphous solid. **Lysergine ((+)-1a)**: ^1H NMR (400 MHz, $CDCl_3$) δ 7.86 (s, 1H), 7.22 – 7.12 (m, 3H), 6.90 (s, 1H), 6.32 (s, 1H), 3.53 (dd, *J* = 14.5, 5.5 Hz, 1H), 3.12 – 3.04 (m, 1H), 2.99 (dd, *J* = 11.0, 5.5 Hz, 1H), 2.83 – 2.74 (m, 1H), 2.68 (ddd, *J* = 14.5, 11.5, 2.0 Hz, 1H), 2.56 (s, 3H), 2.15 (t, *J* = 10.5 Hz, 1H), 1.07 (d, *J* = 7.0 Hz, 3H). $[\alpha]_D^{20}$ = + 61,4 (c = 0.044, pyridine). HRMS (ESI-TOF) m/z: $[M + H]^+$ calcd for $C_{16}H_{19}N_2$, 239.1543; found, 239.1540. The chemical–physical data are in according to the compound reported in the literature Isolysergine.[3,4] **Isolysergine ((+)-1b):** ^1H NMR (400 MHz, $CDCl_3$) δ 7.84 (s, 1H), 7.18 (d, *J* = 7.5 Hz, 1H), 7.16 – 7.11 (m, 2H), 6.90 (s, 1H), 6.39 (dd, *J* = 4.5, 1.5 Hz, 1H), 3.43 (dd, *J* = 14.5, 5.5 Hz, 1H) 3.25 – 3.20 (m, 1H) 2.77 – 2.68 (m, 2H), 2.62 (dd, *J* = 4.0, 1.5 Hz, 1H), 2.55 (s, 3H), 2.55 – 2.44 (m, 1H), 1.19 (d, *J* = 7.0 Hz, 3H); $[\alpha]_D^{20}$ = + 196 (c = 0.055, pyridine). HRMS (ESI-TOF) m/z: $[M + H]^+$ calcd for $C_{16}H_{19}N_2$, 239.1543; found, 239.1541. The chemical–physical data are in according to the compound reported in the literature.[5,6]

(6a*R*,9*R*)-9-(Methoxymethyl)-4,6,6a,7,8,9-hexahydroindolo[4,3-*fg*]quinoline ((+)-13))

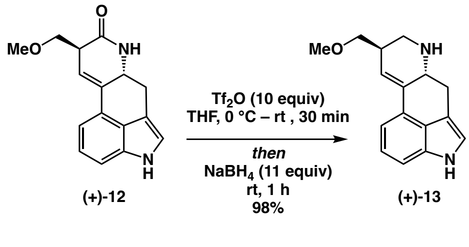

To a solution of compound **(+)-12** (16 mg, 0.06 mmol) in THF (1.2 mL), at 0 °C under N_2, Tf_2O (0.062 mL, 0.6 mmol) was added slowly. The reaction was then allowed to warm to room temperature and stirred for 30 minutes. The reaction was cooled to 0 °C, after which $NaBH_4$ (25 mg, 0.66 mmol) was added portion-wise. The reaction was then stirred at room temperature for 1 hour and quenched with 2 M Na_2CO_3 solution. The mixture was extracted four times with EtOAc. The combined organic phases were washed with brine, dried over Na_2SO_4, filtered, and the solvent

evaporated under reduced pressure. The yellowish gel crude residue was purified by flash-chromatography (DCM/MeOH 9:1) to give **(+)-13** (15 mg, 98%) as yellowish amorphous solid. ^{1}H NMR (400 MHz, $CDCl_3$/ MeOH-$d_4$10/1) δ 7.24 (d, *J* = 8 Hz, 1H), 7.14 – 7.12 (m, 2H), 6.92 (s, 1H), 6.37 (s, 1H), 4.18 – 4.15 (m, 1H), 3.62 – 3.39 (m, 5H), 3.35 (s, 3H), 2.99 – 2.93 (m, 2H). ^{13}C{1H} NMR (100 MHz, $CDCl_3$/ MeOH-$d_4$10/1): δ 134.1, 131.8, 125.9, 125.1, 123.1, 120.2, 119.6, 112.3, 110.9, 106.9, 73.7, 59.2, 53.5, 44.0, 34.0, 26.7; $[\alpha]_D^{20}$ = +58,6 (c = 0.284, MeOH). HRMS (ESI-TOF) m/z: $[M + H]^+$ calcd for $C_{16}H_{19}N_2O$, 255.1492; found, 255.1488.

(6a*R*,9*R*)-9-(Methoxymethyl)-7-methyl-4,6,6a,7,8,9-hexahydroindolo[4,3-*fg*]quinoline ((+)-14)

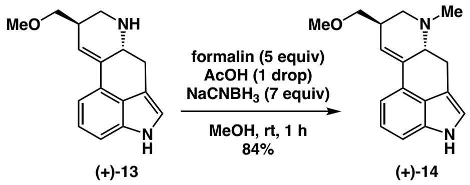

To a solution of **(+)-13** (10 mg, 0.04 mmol) in MeOH (1 mL) were added formalin (0.050 mL, 0.2 mmol), AcOH (1 drop), and $NaCNBH_3$ (17.3 mg, 0.28 mmol). The reaction mixture was stirred at room temperature for 1 hour. The solution was then diluted with EtOAc and a saturated solution of Na_2CO_3. The layers were separated, and the aqueous phase was extracted four times with EtOAc. The combined organic phases were washed with brine, dried over Na_2SO_4, filtered, and the solvent evaporated under reduced pressure. The crude product was purified by preparative TLC (DCM/MeOH, 95:5) to yield **(+)-14** (9 mg, 84%) as off-white amorphous solid. ^{1}H NMR (400 MHz, $CDCl_3$) δ 7.94 (s, 1H), 7.23 – 7.12 (m, 3H), 6.90 (s, 1H), 6.35 (s, 1H), 3.53 (dd, *J* = 14.5, 5.5 Hz, 1H), 3.46 (dd, *J* = 9.5, 6.0 Hz, 1H), 3.41 (s, 3H), 3.36 (dd, *J* = 9.5, 8.0 Hz, 1H), 3.16 – 3.09 (m, 2H), 3.08 – 2.98 (m, 1H), 2.70 (ddd, *J* = 14.5, 11.5, 2.0 Hz, 1H), 2.58 (s, 3H), 2.27 (t, *J* = 10.5 Hz, 1H). ^{13}C{1H} NMR (100 MHz, $CDCl_3$): δ 135.6, 134.1, 128.7, 126.3, 123.4, 121.9, 118.2, 112.3, 111.4, 109.4, 75.5, 63.5, 59.1, 57.5, 44.1, 36.7, 27.5; $[\alpha]_D^{20}$ = +44,5 (c = 0.068, $CHCl_3$). HRMS (ESI-TOF) m/z: $[M + H]^+$ calcd for $C_{17}H_{21}N_2O$, 269.1648; found, 269.1643.

(+)-Lysergol ((+)-2)

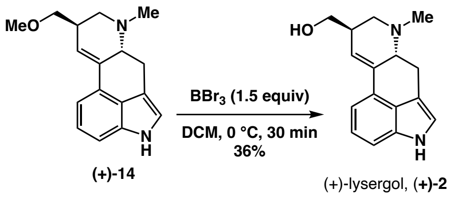

To a solution of **(+)-14** (10 mg, 0.038 mmol) in DCM (0.38 mL), at 0 °C under N_2, was added dropwise a 1 M solution of BBr_3 (0.057 mL, 0.057 mmol). The reaction mixture was stirred at 0 °C

for 30 minutes. Then was quenched with a saturated solution of Na_2CO_3 and extracted four times with a 9:1 mixture of DCM:MeOH. The combined organic phases were washed with brine, dried over Na_2SO_4, filtered, and the solvent evaporated under reduced pressure. The crude product was purified by preparative TLC (DCM/MeOH, 9:1) to yield **(+)-2** (4 mg, 36%) as off-white amorphous solid. ^{1}H NMR (400 MHz, MeOH-d_4) δ 7.17 (dd, J = 7.5, 1.0 Hz, 1H), 7.13 – 7.04 (m, 2H), 6.94 (d, J = 1.5 Hz, 1H), 6.40 (s, 1H), 3.63 (dd, J = 11.0, 6.0 Hz, 1H), 3.58 – 3.51 (m, 2H), 3.15 – 3.10 (m, 2H), 2.91 – 2.82 (m, 1H), 2.65 (dd, J = 14.5, 11.5, 1.5 Hz, 1H), 2.58 (s, 3H), 2.28 (t, J = 11.0 Hz, 1H); $[\alpha]_D^{20}$ = +62,5 (c = 0.12, MeOH). The chemical–physical data are in according to the compound reported in the literature. HRMS (ESI-TOF) m/z: $[M + H]^+$ calcd for $C_{16}H_{19}N_2O$, 255.1492; found, 255.1495.[7]

Table S1. Comparison of ^{1}H NMR data of lysergine (**(+)-1a**) with literature values

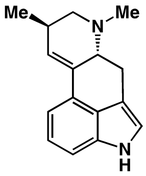

(+)-lysergine, **(+)-1a**

**This work**<br><br>^{1}H NMR (400 MHz $CDCl_3$)	**Rebek et al. Report**[3]<br><br>^{1}H NMR (300 MHz, $CDCl_3$)
7.86 (s, 1H)	7.95 (br s, 1H)
7.22 – 7.12 (m, 3H)	7.17 – 7.21 (m, 3H)
6.90 (s, 1H)	6.91 (m, 1H)
6.32 (s, 1H)	6.34 (br s, 1H)
3.53 (dd, *J* = 14.5, 5.5 Hz, 1H)	3.55 (dd, *J* = 14.5, 5.5 Hz, 1H)
3.12 – 3.04 (m, 1H)	3.20 – 3.29 (m, 1H)
2.99 (dd, *J* = 11.0, 5.5 Hz, 1H)	3.08 (dd, *J* = 11.0, 5.2 Hz, 1H)
2.83 – 2.74 (m, 1H)	2.77 (m, 1H)
2.68 (ddd, *J* = 14.5, 11.5, 2.0 Hz, 1H)	2.76 (ddd, *J* = 14.0, 11.0, 1.8 Hz)
2.56 (s, 3H)	2.60 (s, 3H)
2.15 (t, *J* = 10.5 Hz, 1H)	2.20 (t, *J* = 11.0 Hz, 1H)
1.07 (d, *J* = 7.0 Hz, 3H)	1.09 (d, *J* = 7.1 Hz, 3H)

Table S2. Comparison of ^{1}H NMR data of isolysergine (**(+)-1b**) with literature values

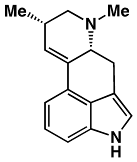

(+)-isolysergine, **(+)-1b**

**This work**<br><br>^{1}H NMR (400 MHz CDCl$_3$)	**Wipf et al. Reported**[5]<br><br>^{1}H NMR (500 MHz CDCl$_3$)
7.84 (s, 1H)	7.86 (br s, 1 H)
7.18 (d, *J* = 7.5 Hz, 1H)	7.19 (d, *J* = 8.0 Hz, 1H)
7.16 – 7.11 (m, 2H)	7.16 (app t, *J* = 7.3 Hz, 1H)
-	7.12 (d, *J* = 6.5 Hz, 1H)
6.90 (s, 1H)	6.90 (s, 1H)
6.39 (dd, *J* = 4.5, 1.5 Hz, 1H)	6.39 (dd, *J* = 4.0, 1.0 Hz, 1H)
3.43 (dd, *J* =14.5, 5.5 Hz, 1H)	3.44 (dd, *J* = 8.5, 4.0 Hz, 1H)
3.25 – 3.20 (m, 1H)	3.30 – 3.23 (m, 1H)
2.77 – 2.68 (m, 2H)	2.78 – 2.73 (m, 2H)
2.62 (dd, *J* = 4.0, 1.5, Hz, 1H)	2.64 (dd, *J* = 3.5, 1.5 Hz, 1H)
2.55 (s, 3H)	2.57 (s, 3H)
2.55 – 2.44 (m, 1H)	2.55– 2.49 (m, 1H)
1.19 (d, *J* = 7.0 Hz, 3H)	1.20 (d, *J* = 7.0 Hz, 3H)

Table S3. Comparison of ^{1}H NMR data of lysergol (**(+)-2**) with literature values.

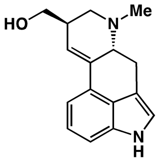

(+)-lysergol, **(+)-2**

**This work**<br><br>^{1}H NMR (400 MHz CD_3OD)	**Wipf et al. Reported**[7]<br><br>^{1}H NMR (500 MHz CD_3OD)
7.17 (dd, *J* = 7.5, 1.0 Hz, 1H)	7.17 (d, *J* = 7.5 Hz, 1H)
7.13 – 7.04 (m, 2H)	7.12 – 7.08 (m, 2H)
6.94 (d, *J* = 1.5 Hz, 1H)	6.95 (d, *J* = 1.5, 1H)
6.40 (s, 1H)	6.41 (s, 1H)
3.63 (dd, *J* = 11.0, 6.0 Hz, 1H)	3.64 (dd, *J* = 10.8, 5.8 Hz, 1H)
3.58 – 3.51 (m, 2H)	3.59 – 3.52 (m, 2H)
3.15 – 3.10 (m, 2H)	3.21 – 3.15 (m, 2H)
2.91 – 2.82 (m, 1H)	2.89 – 2.87 (m, 1H)
2.65 (dd, *J* = 14.5, 11.5, 1.5 Hz, 1H)	2.67 (ddd, *J* = 14.5, 11.5, 1.5 Hz, 1H)
2.58 (s, 3H)	2.62 (s, 3 H)
2.28 (t, *J* = 11.0 Hz, 1H)	2.35 (t, *J* = 11.0 Hz, 1H)

References

[1] F. Bartoccini, A. Regni, M. Retini, G. Piersanti. Concise Catalytic Asymmetric Synthesis of (R)-4-Amino Uhle's Ketone. *Org. Biomol. Chem.* **2021**, *19*, 2932 – 2940.

[2] F. Bartoccini, A. Regni, M. Retini, G. Piersanti. Asymmetric Total Synthesis of All Rugulovasine Stereoisomers and Preliminary Evaluation of Their Biological Properties. *Eur. J. Org. Chem.* **2022**, *2022* (17), e202200315.

[3] J. Rebek, D. F. Tai, Y. K. Shue. Synthesis of Ergot Alkaloids from Tryptophan. *J. Am. Chem. Soc.* **1984**, *106*, 1813 – 1819.

[4] M. Abe, S. Yamatodani, T. Yamano, M. Kusumoto. Isolation of Lysergol, Lysergene and Lysergine from the Saprophytic Cultures of Ergot Fungi. *Agric Biol Chem* **1961**, *25*, 594 – 596.

[5] N. R. Tasker, P. Wipf. Concise Total Syntheses of Lysergene, Lysergine, Isolysergine and Festuclavine. *Arkivoc* **2024**, 202312120.

[6] E. Schreier. Zur Stereochemie Der Mutterkornalkaloide Vom Agroclavin- Und Elymoclavin-Typus. 46. Mitteilung Über Mutterkornalkaloide. *Helv. Chim. Acta*. **1958**, *41*, 1984 – 1997.

[7] N. R. Tasker, P. Wipf. A Short Synthesis of Ergot Alkaloids and Evaluation of the 5-HT 1/2 Receptor Selectivity of Lysergols and Isolysergols. *Org. Lett.* **2022**, *24*, 7255 – 7259.

Copies of ^{1}H NMR and ^{13}C{1H} NMR spectra

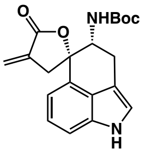

4a

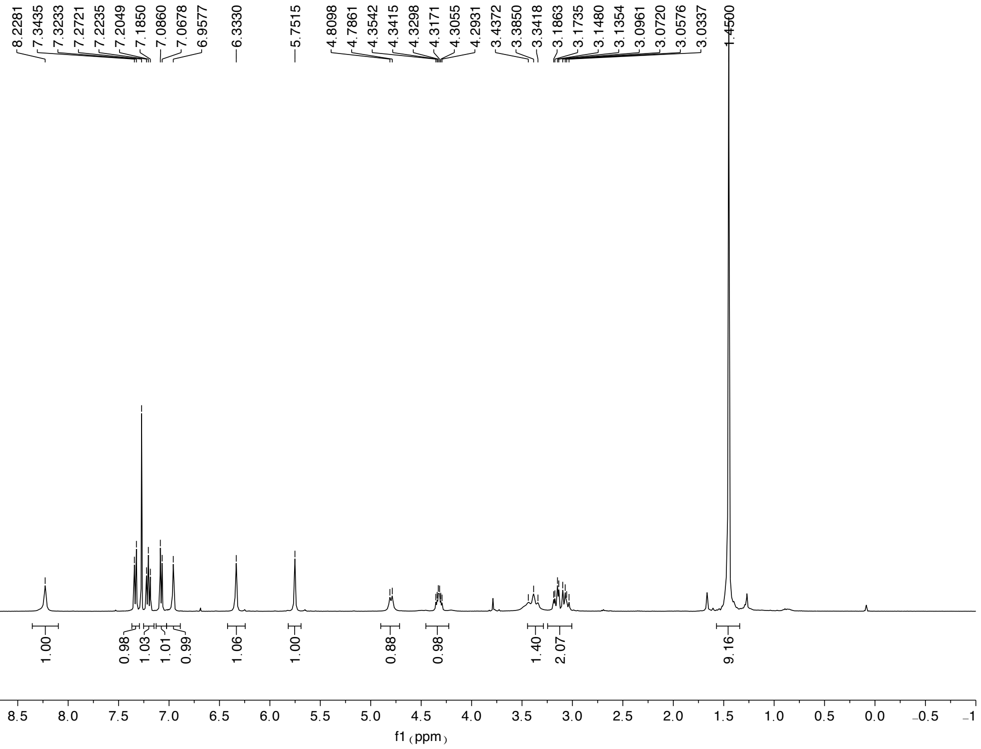

^{1}H NMR spectrum (400 MHz, $CDCl_3$) of compound 4a

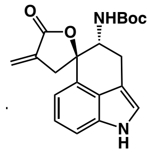

4b

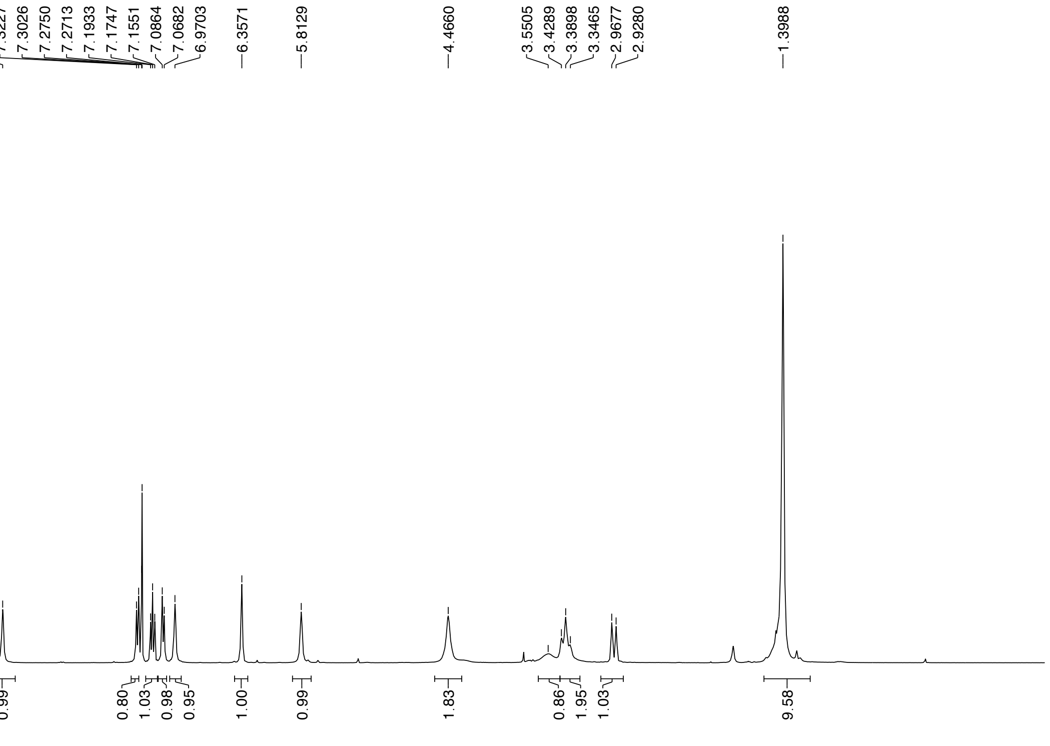

^{1}H NMR spectrum (400 MHz, $CDCl_3$) of compound 4b

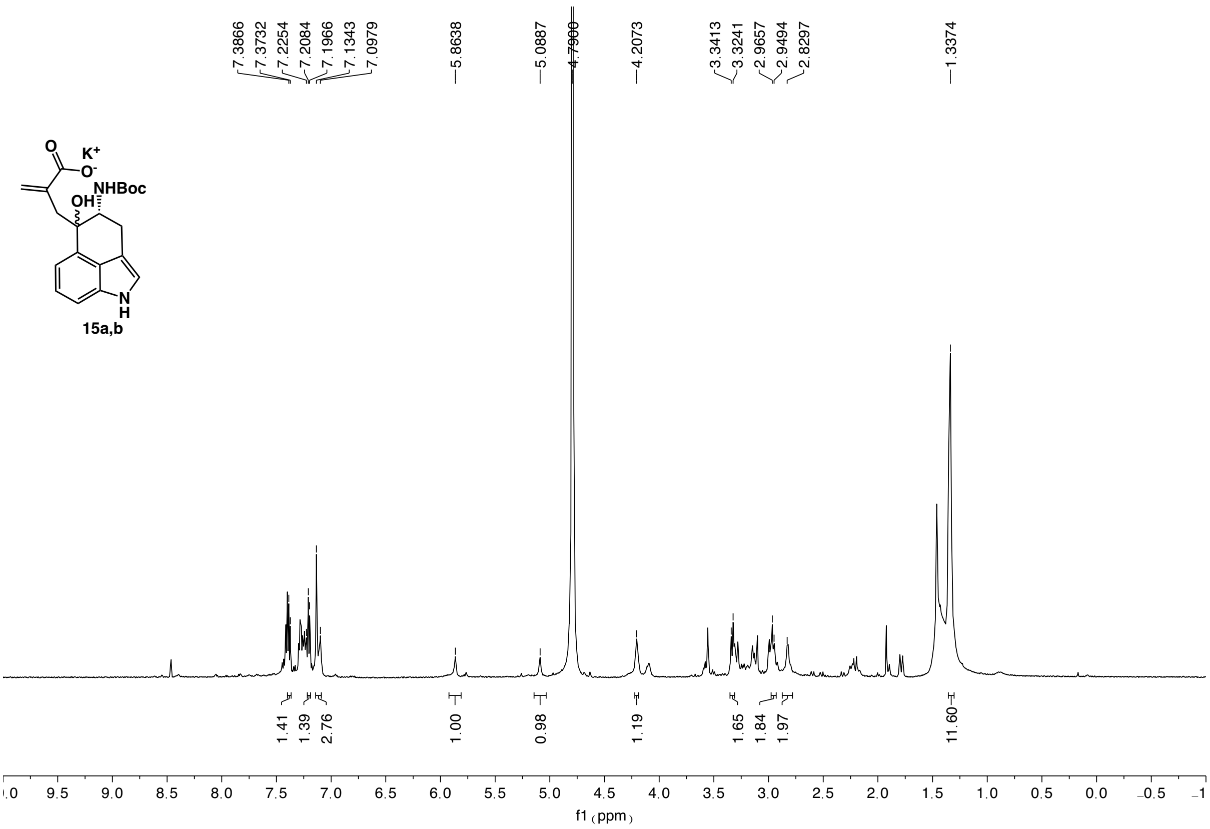

^{1}H NMR spectrum (600 MHz, D_2O) of compound 15a,b

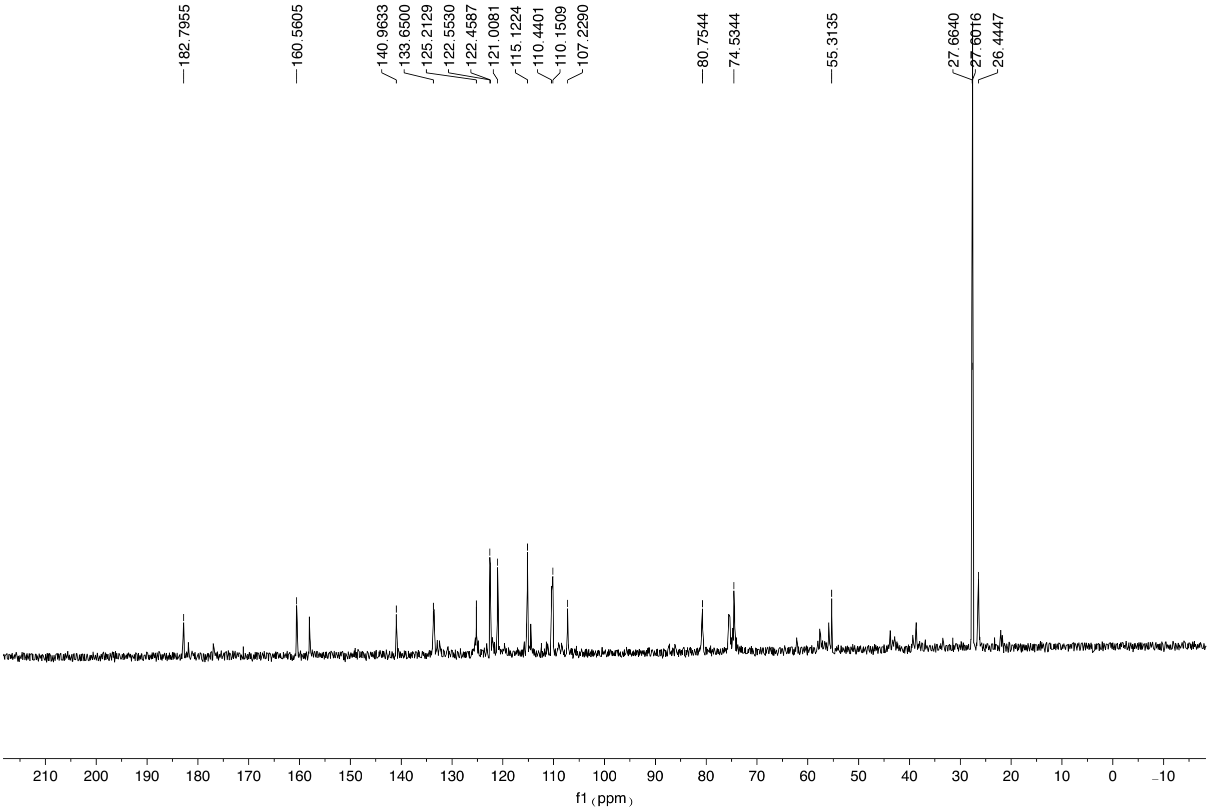

^{13}C{1H} NMR spectrum (150 MHz, D_2O) of compound 15a,b

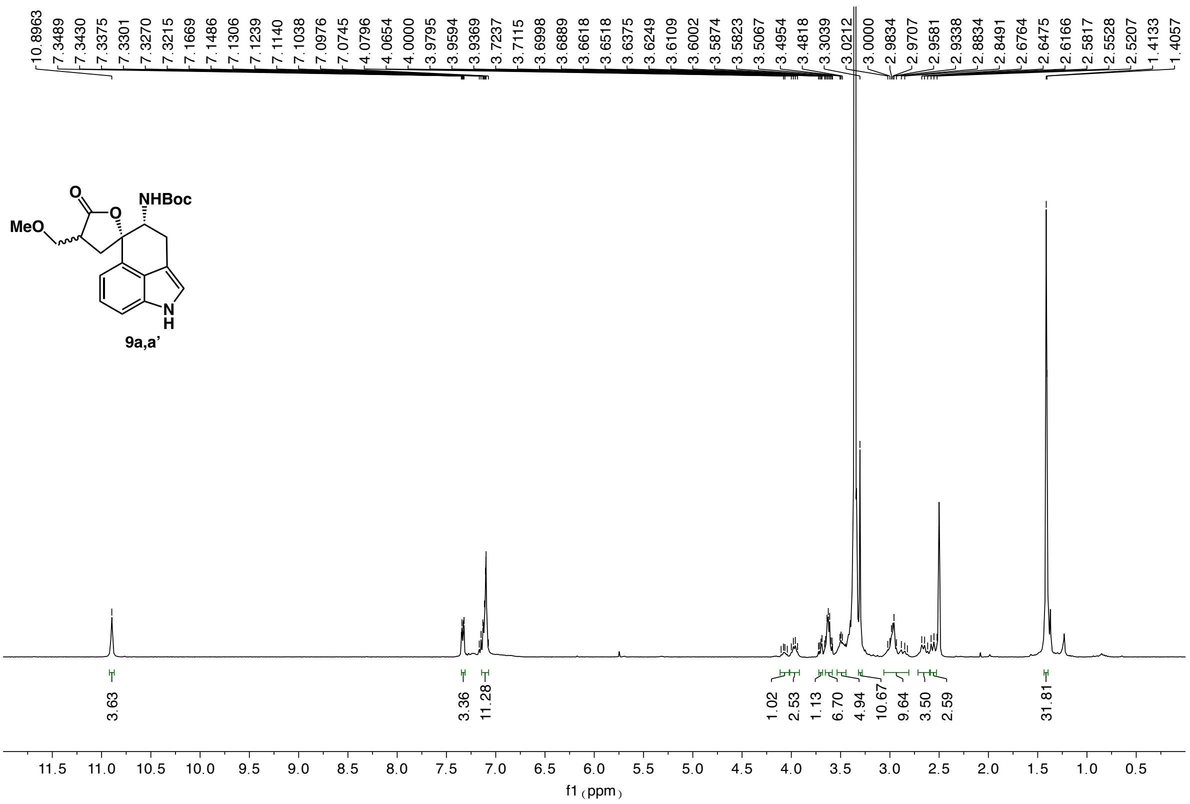

^{1}H NMR spectrum (400 MHz, DMSO-d_6) of compound 9a,a'

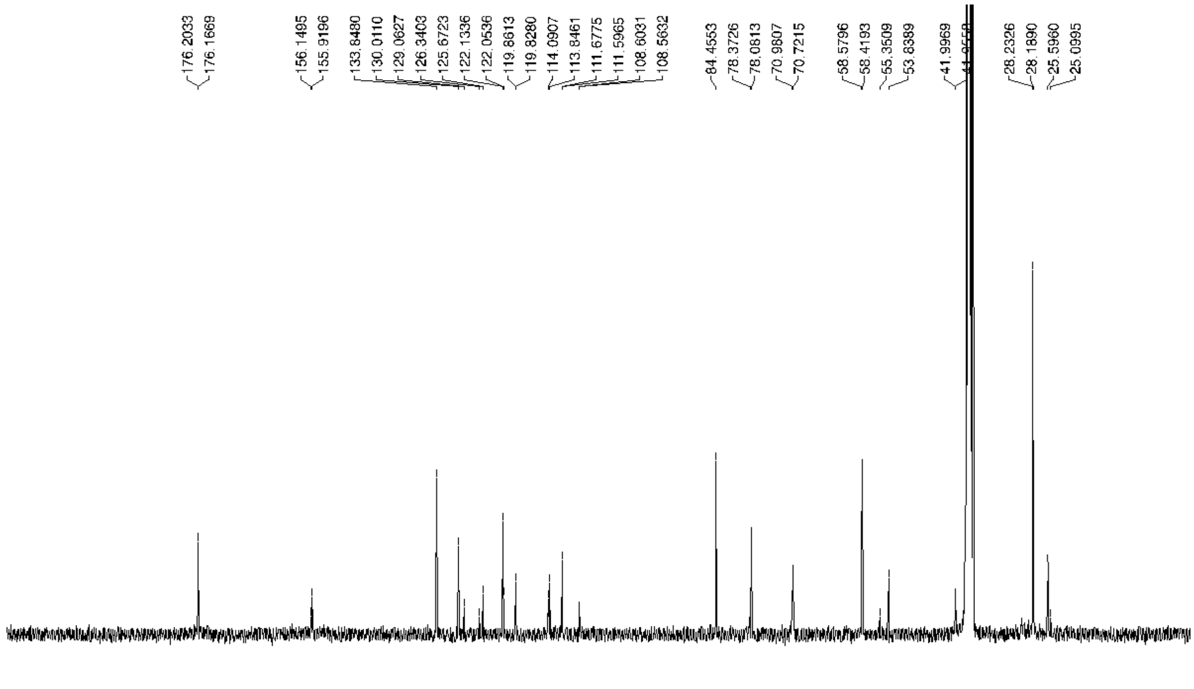

^{13}C{1H} NMR spectrum (100 MHz, DMSO-d_6) of compound 9a,a'

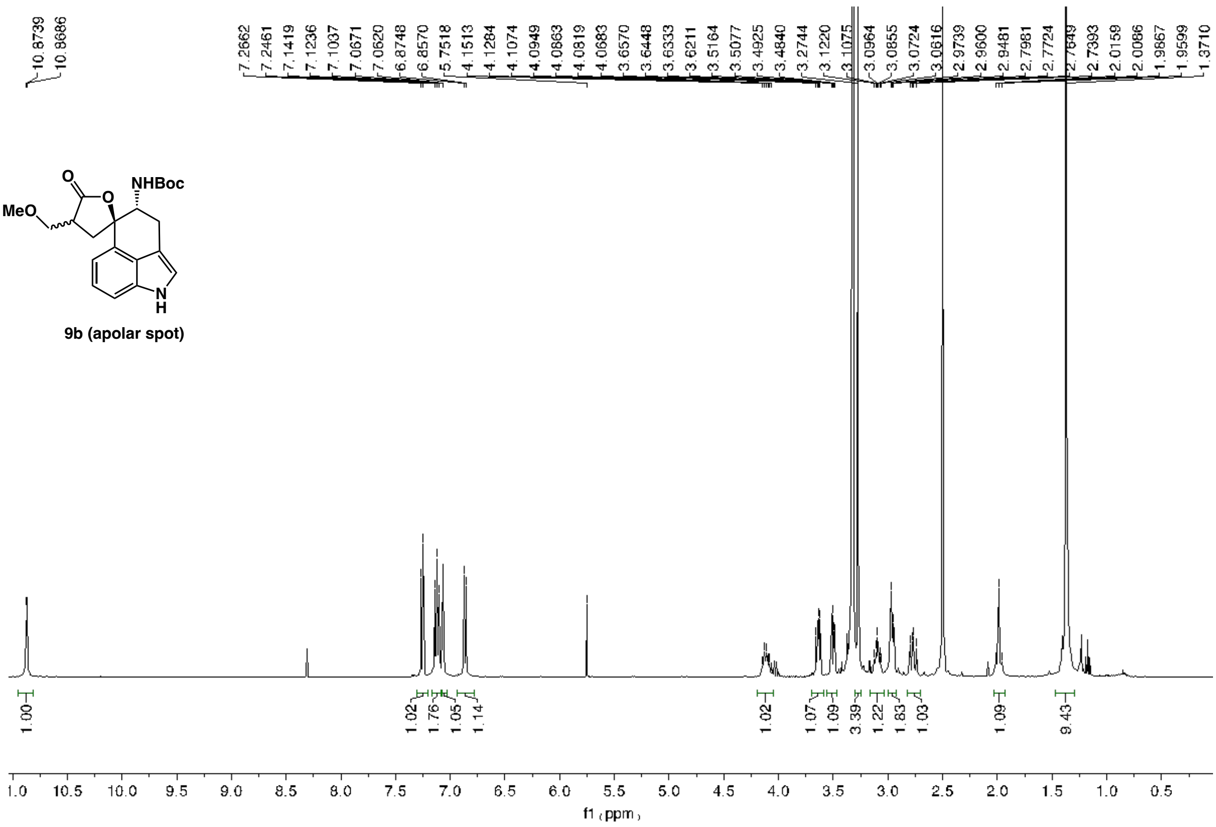

^{1}H NMR spectrum (400 MHz, DMSO-d_6) of compound 9b (apolar spot)

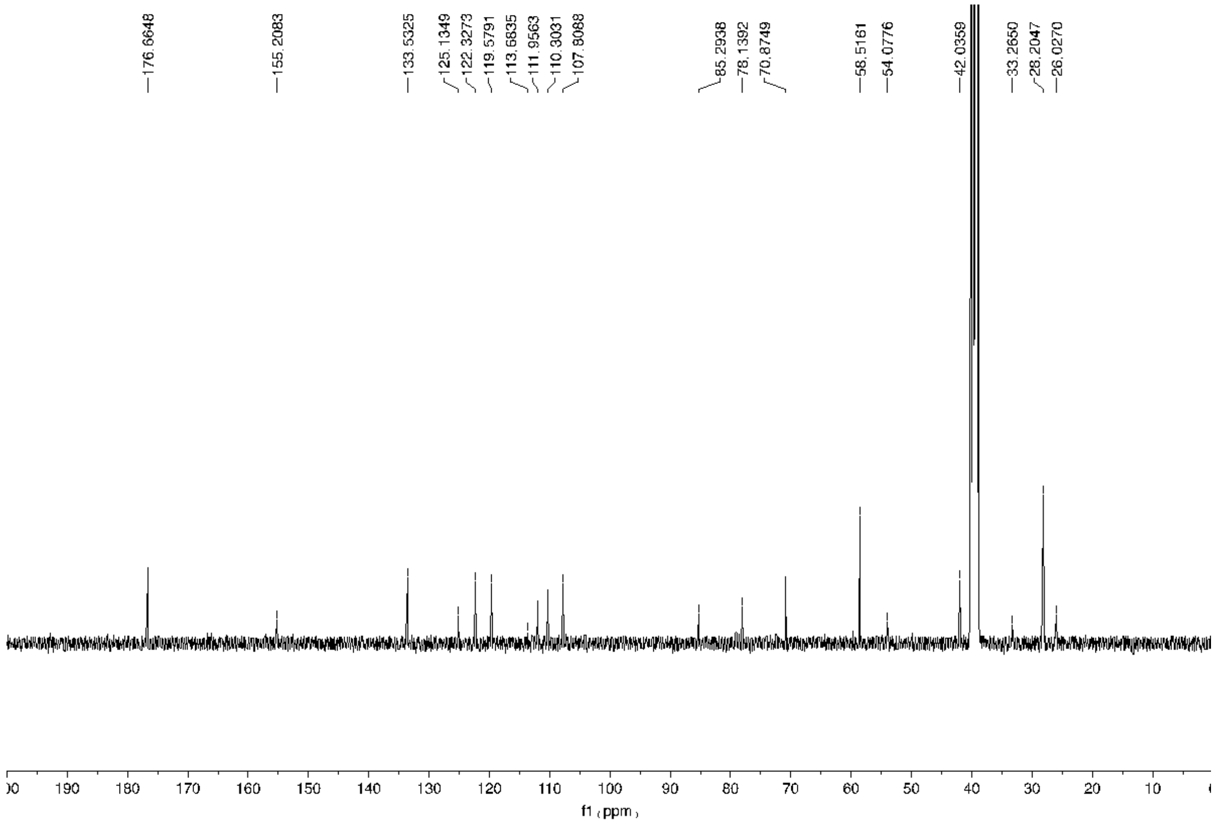

^{13}C{1H} NMR spectrum (100 MHz, DMSO-d_6) of compound 9b (apolar spot)

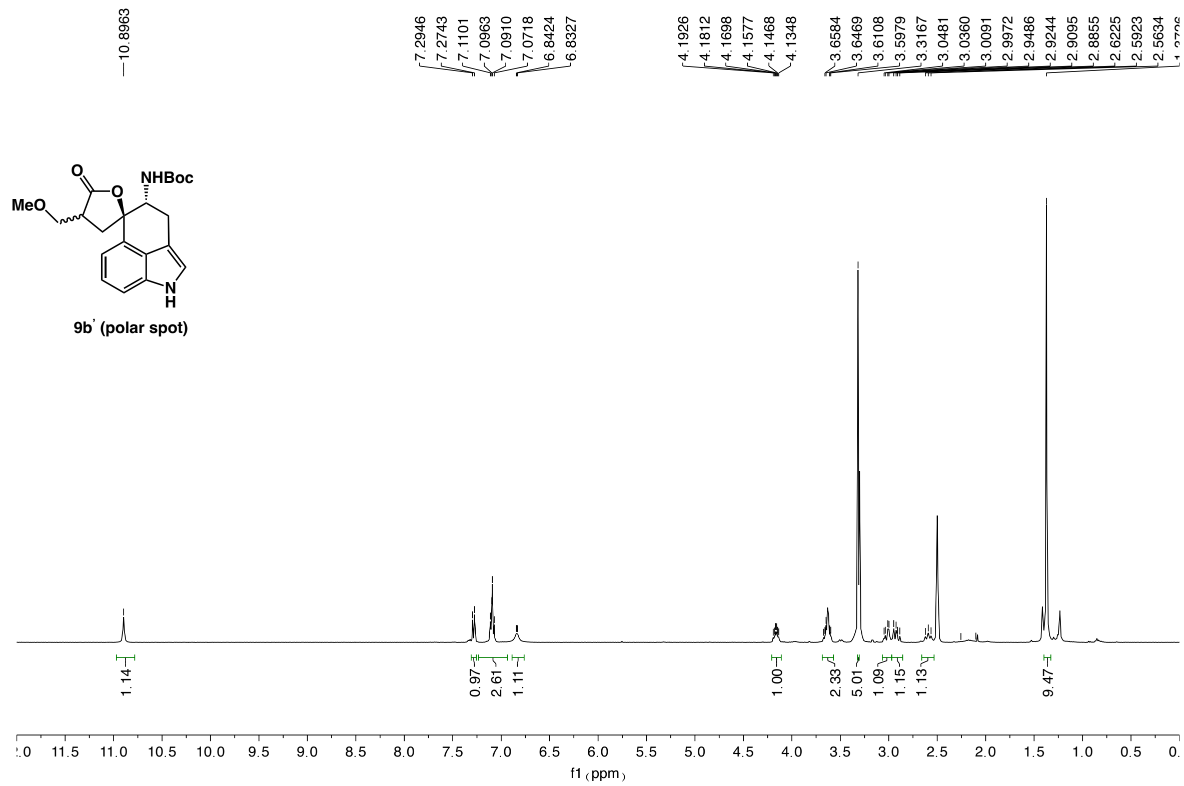

^{1}H NMR spectrum (400 MHz, DMSO-*d₆*) of compound 9b' (polar spot)

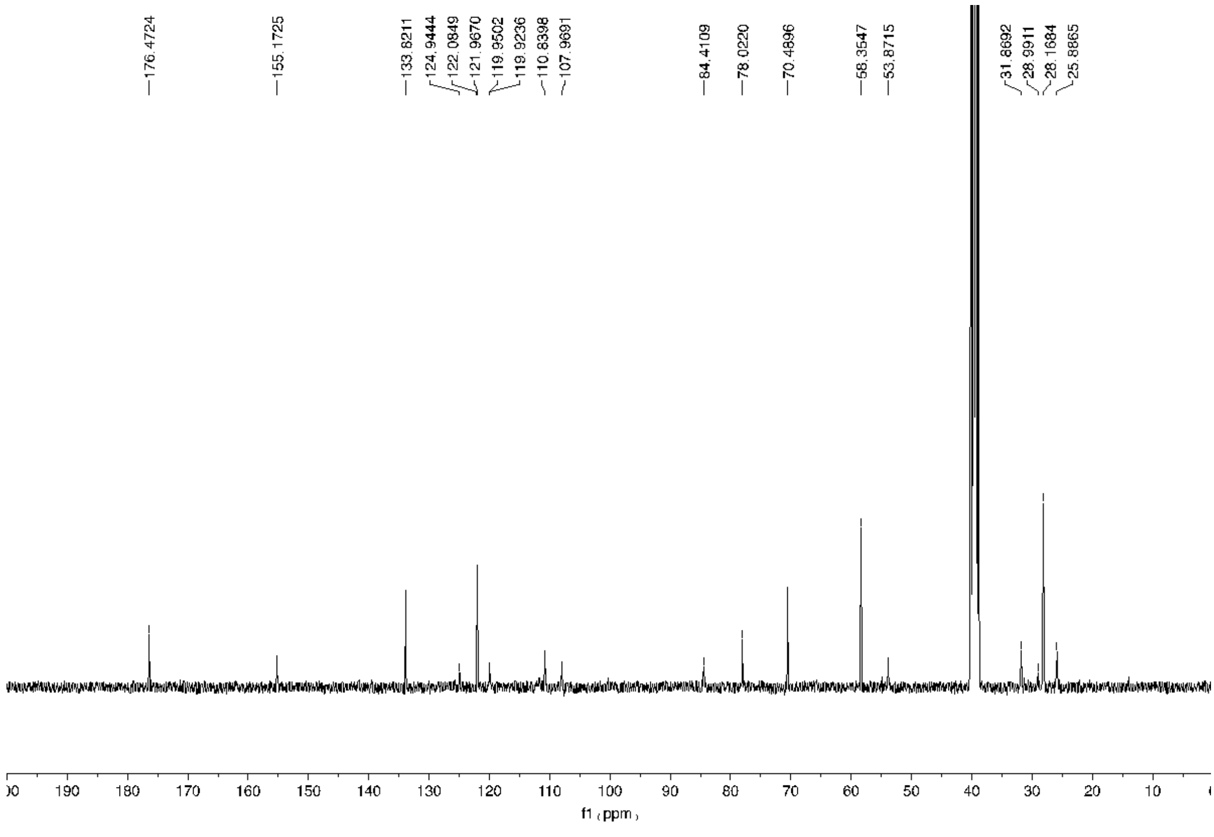

^{13}C{1H} NMR spectrum (100 MHz, DMSO-*d₆*) of compound 9b' (polar spot)

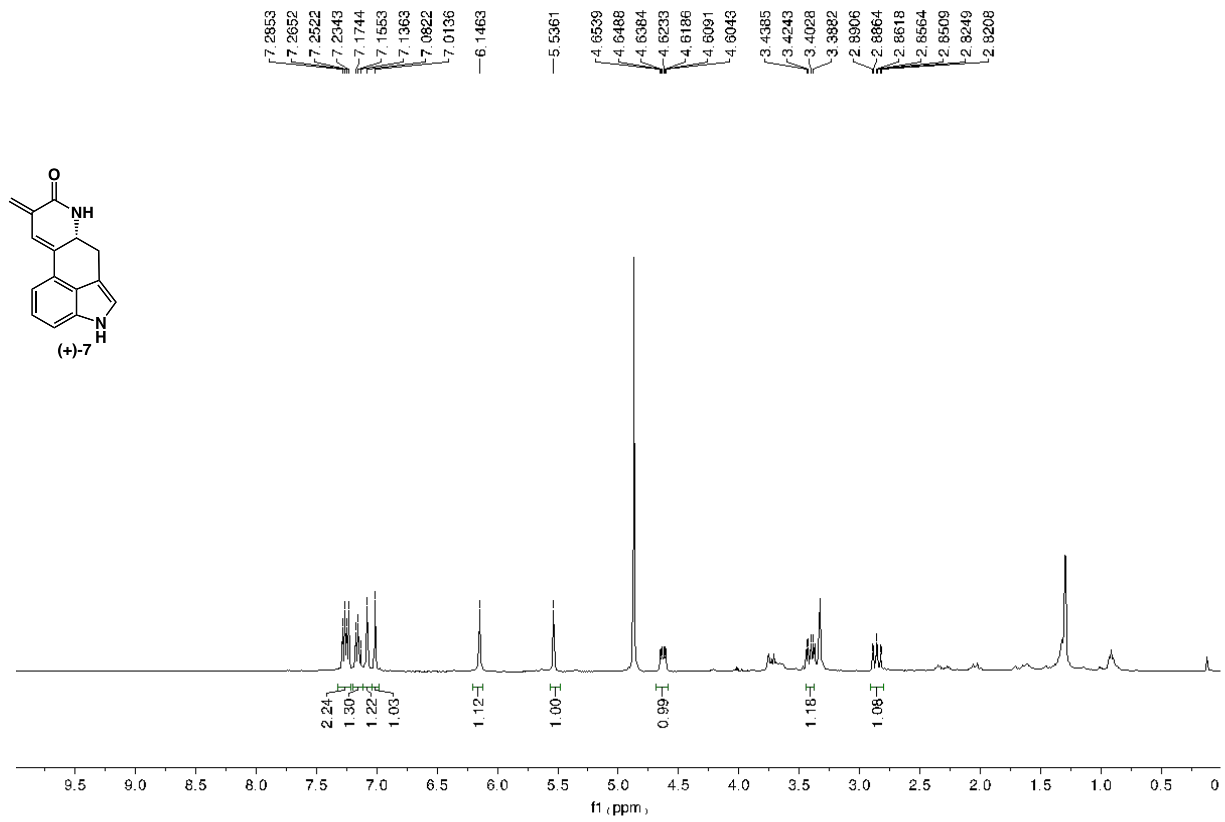

^{1}H NMR spectrum (400 MHz, MeOH-*d*$_4$) of compound (+)-7

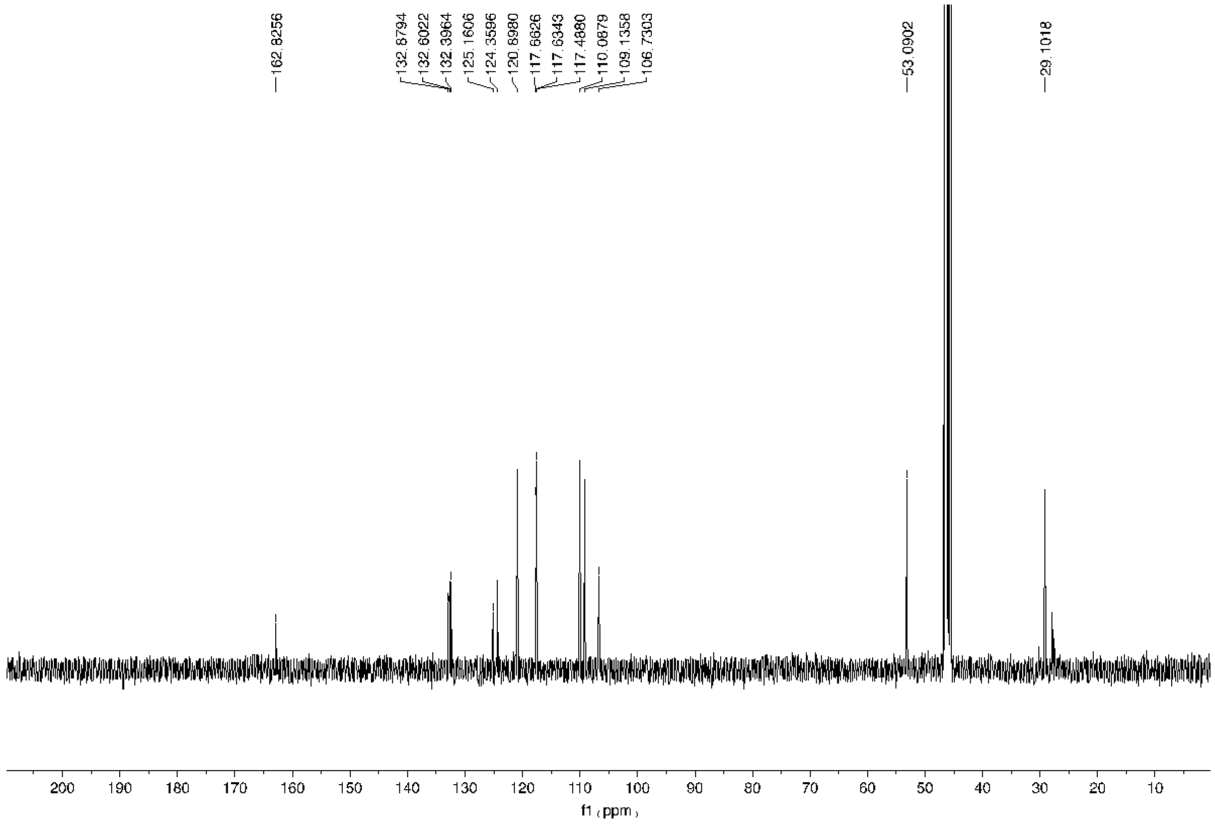

^{13}C{1H} NMR spectrum (100 MHz, MeOH-*d*$_4$) of compound (+)-7

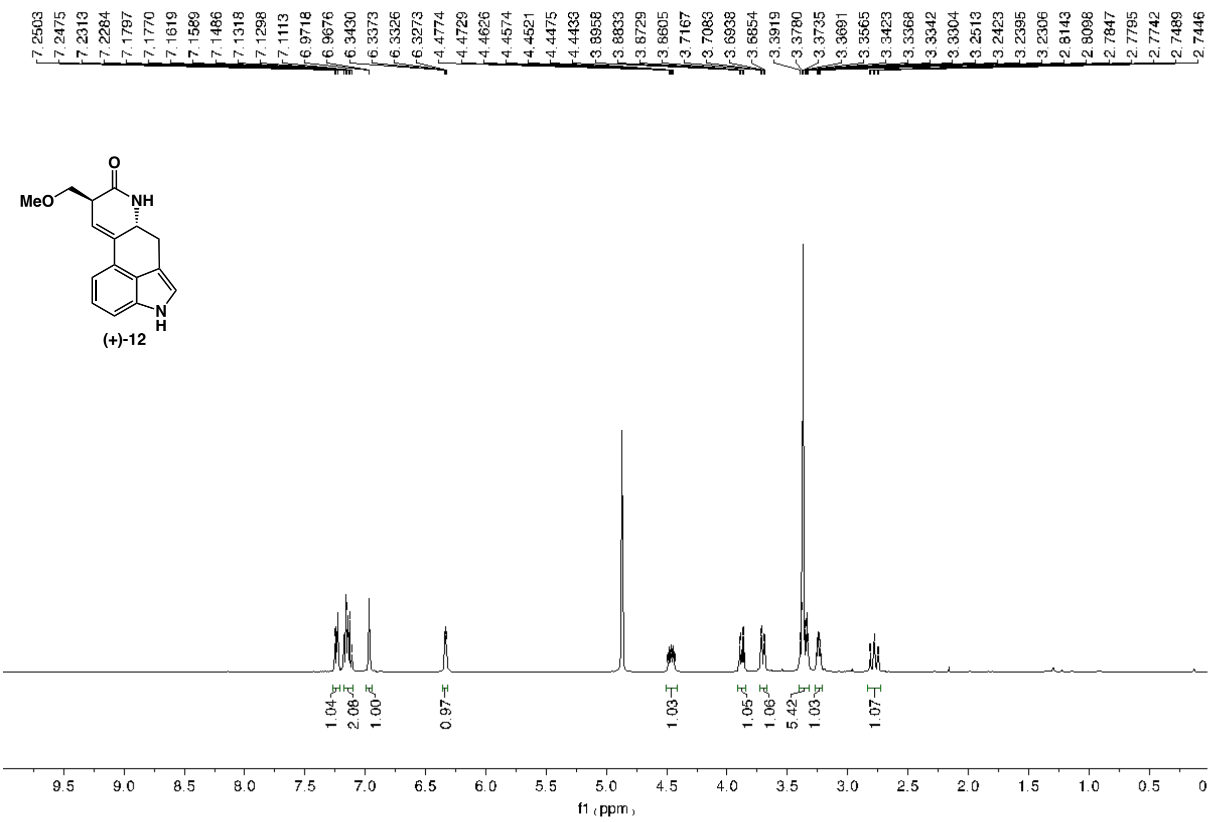

^{1}H NMR spectrum (400 MHz, MeOH-d_4) of compound (+)-12

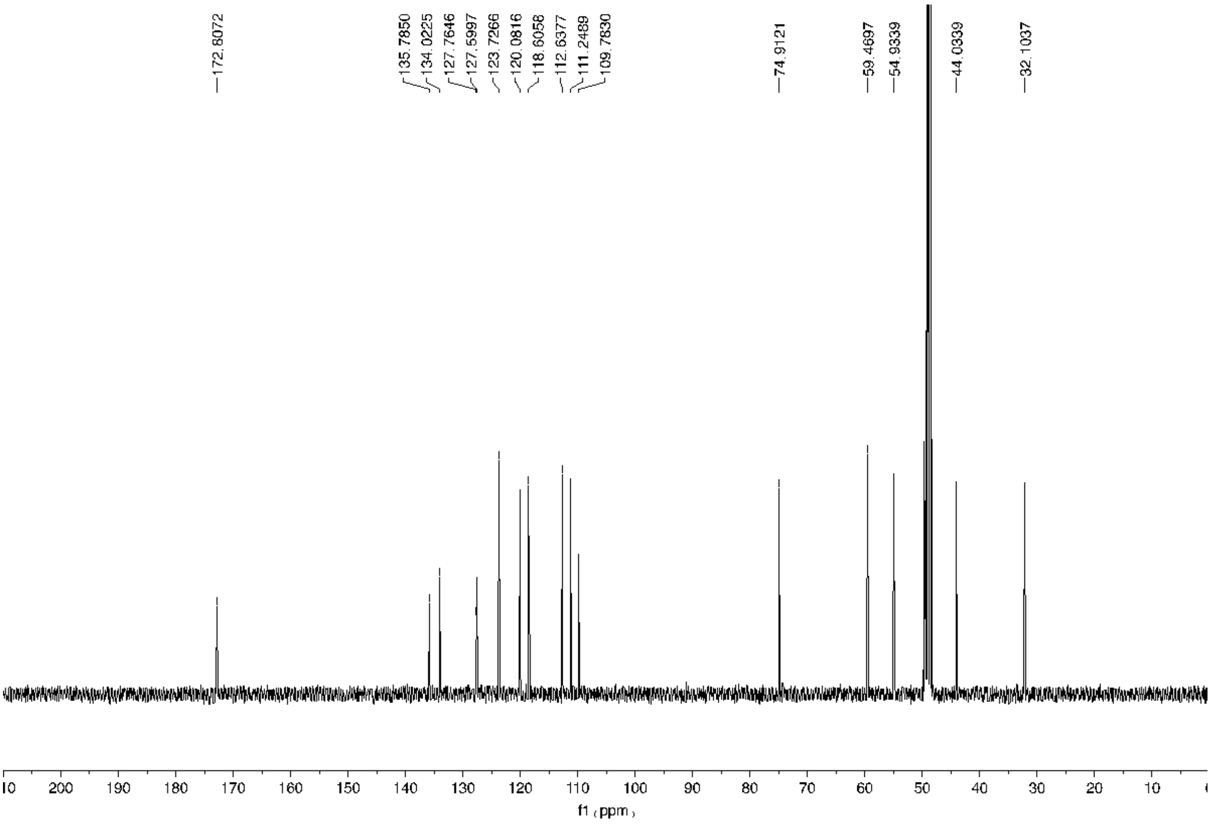

^{13}C{1H} NMR spectrum (100 MHz, MeOH-d_4) of compound (+)-12

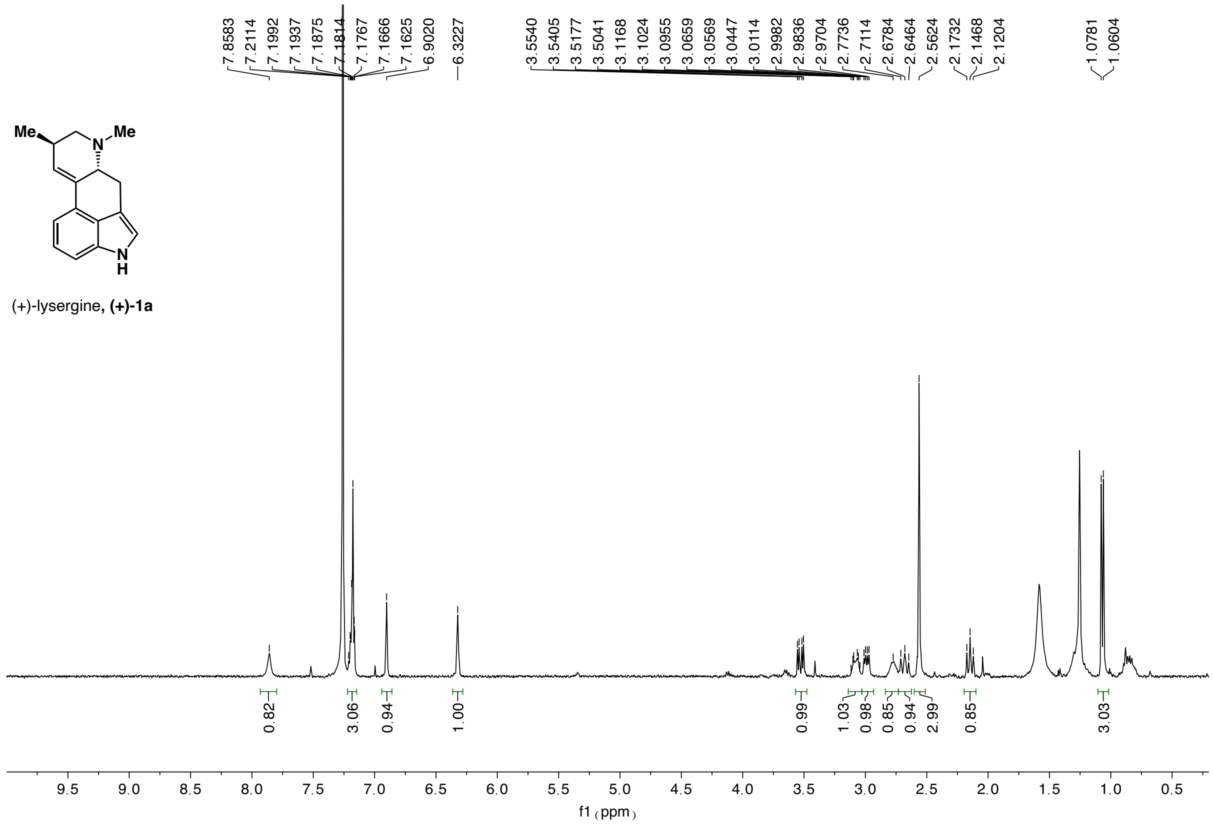

^{1}H NMR spectrum (400 MHz, $CDCl_3$) of compound (+)-1a

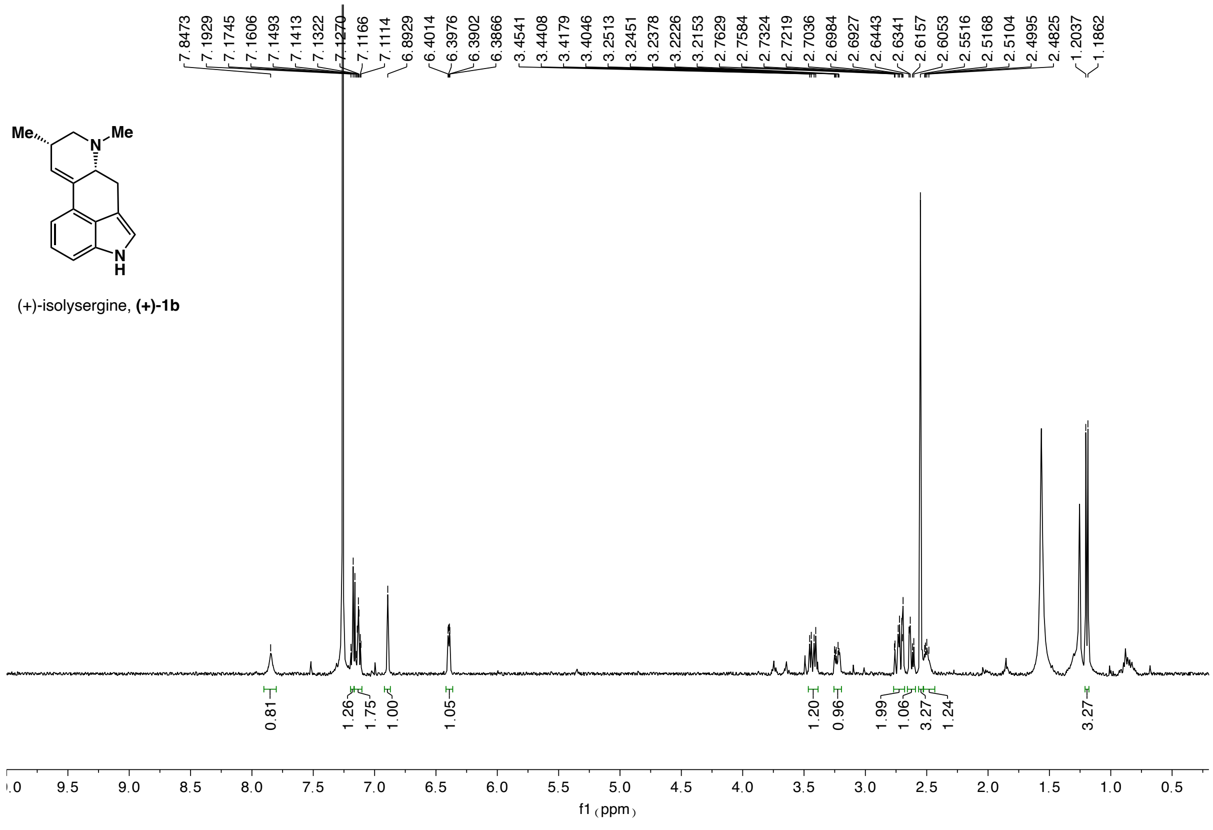

^{1}H NMR spectrum (400 MHz, $CDCl_3$) of compound (+)-1b

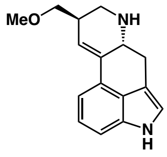

(+)-13

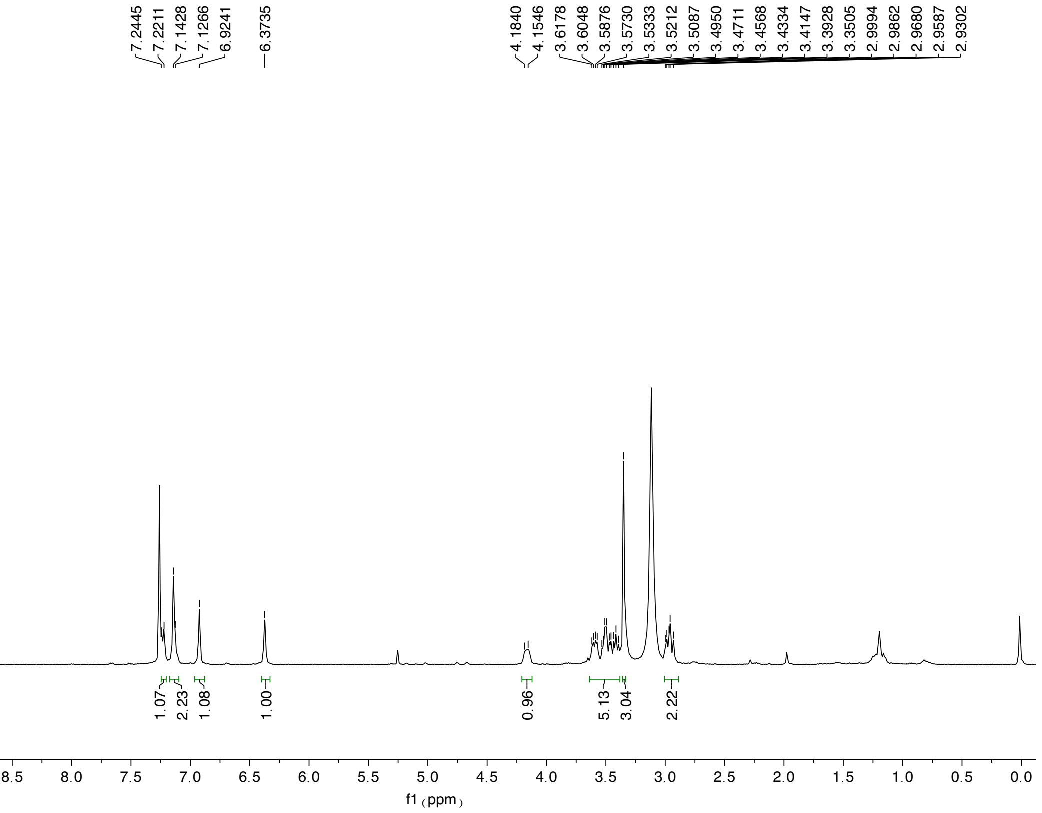

^{1}H NMR spectrum (400 MHz, $CDCl_3$/MeOH-d_4 9:1) of compound (+)-13

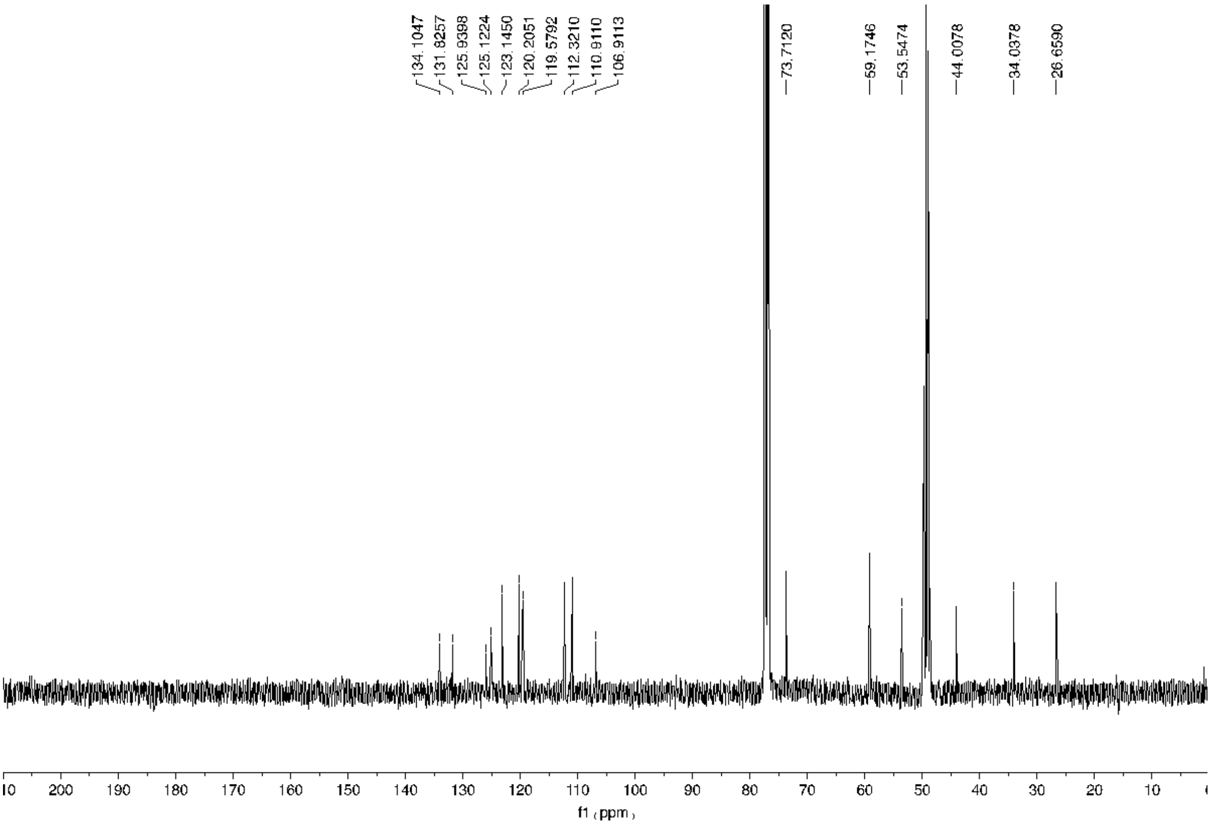

^{13}C{^{1}H} NMR spectrum (100 MHz, $CDCl_3$/MeOH-d_4 9:1) of compound (+)-13

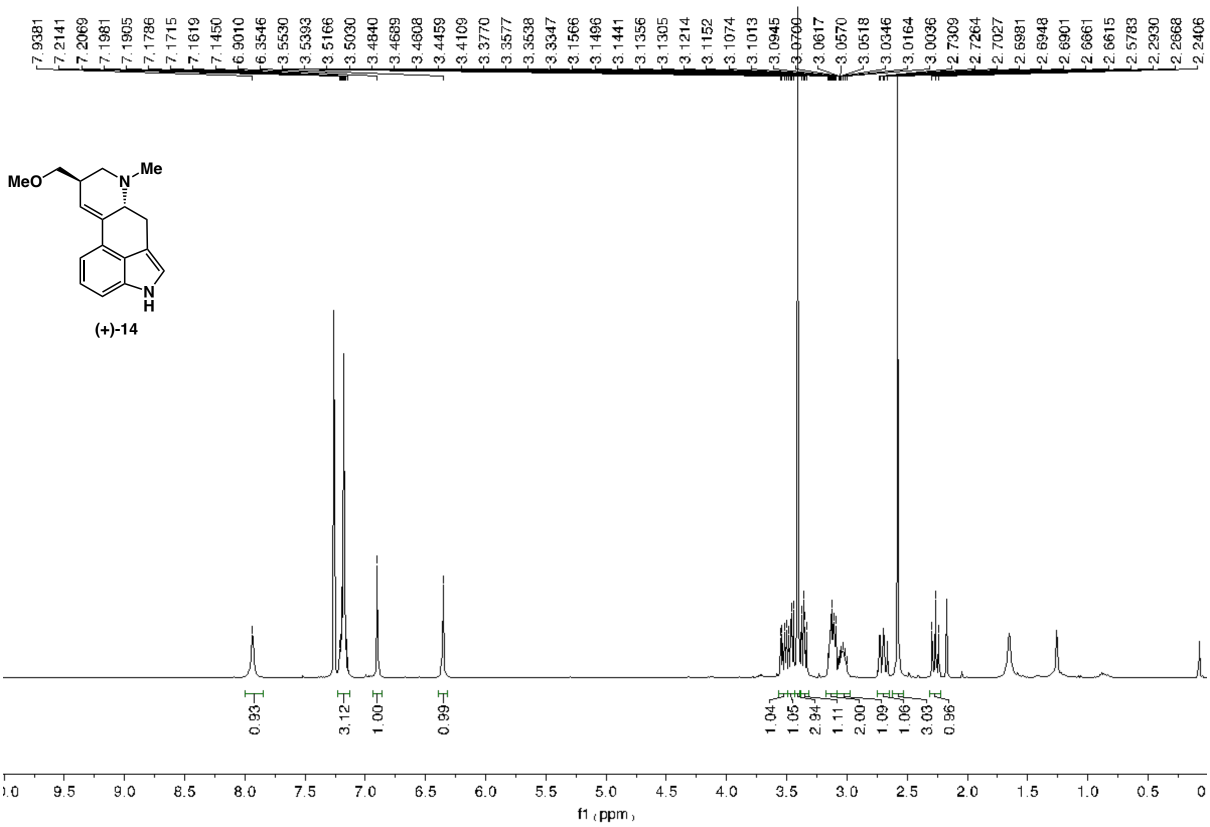

^{1}H NMR spectrum (400 MHz, $CDCl_3$) of compound (+)-14

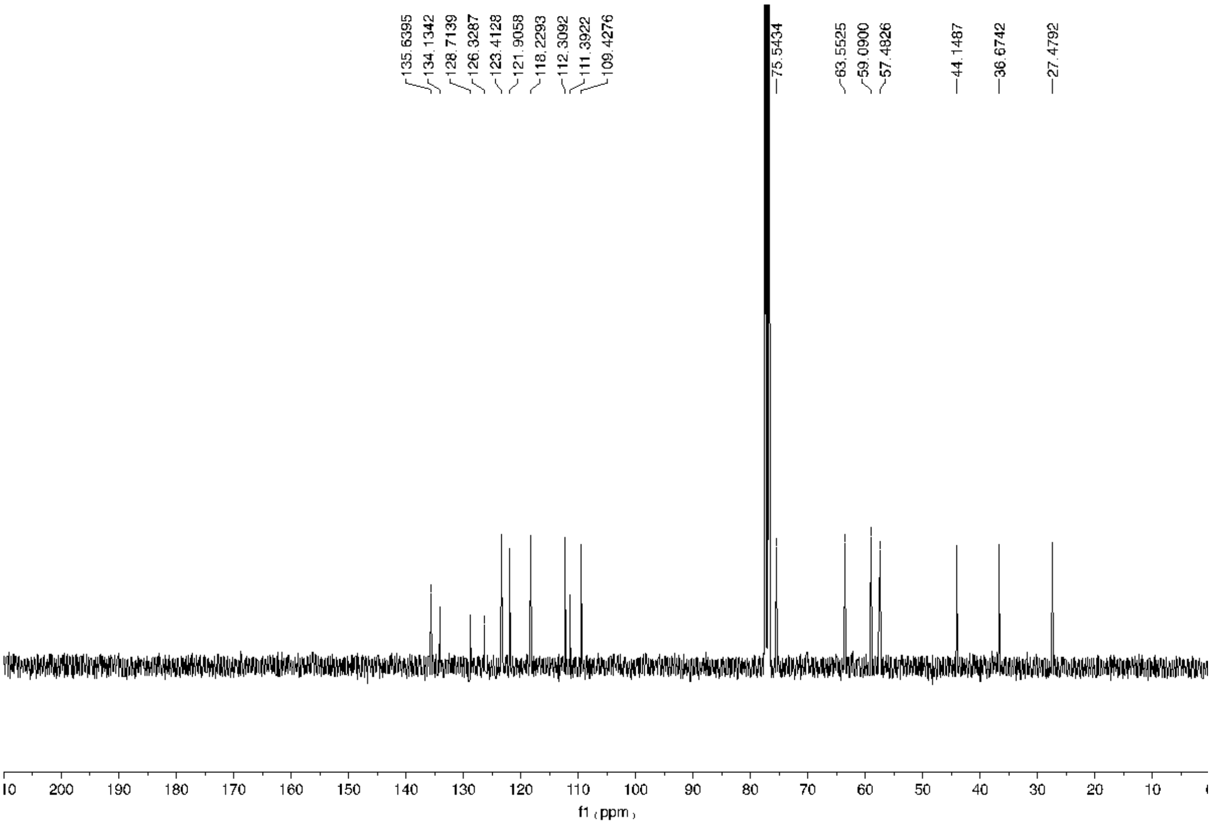

^{13}C{1H} NMR spectrum (100 MHz, $CDCl_3$) of compound (+)-14

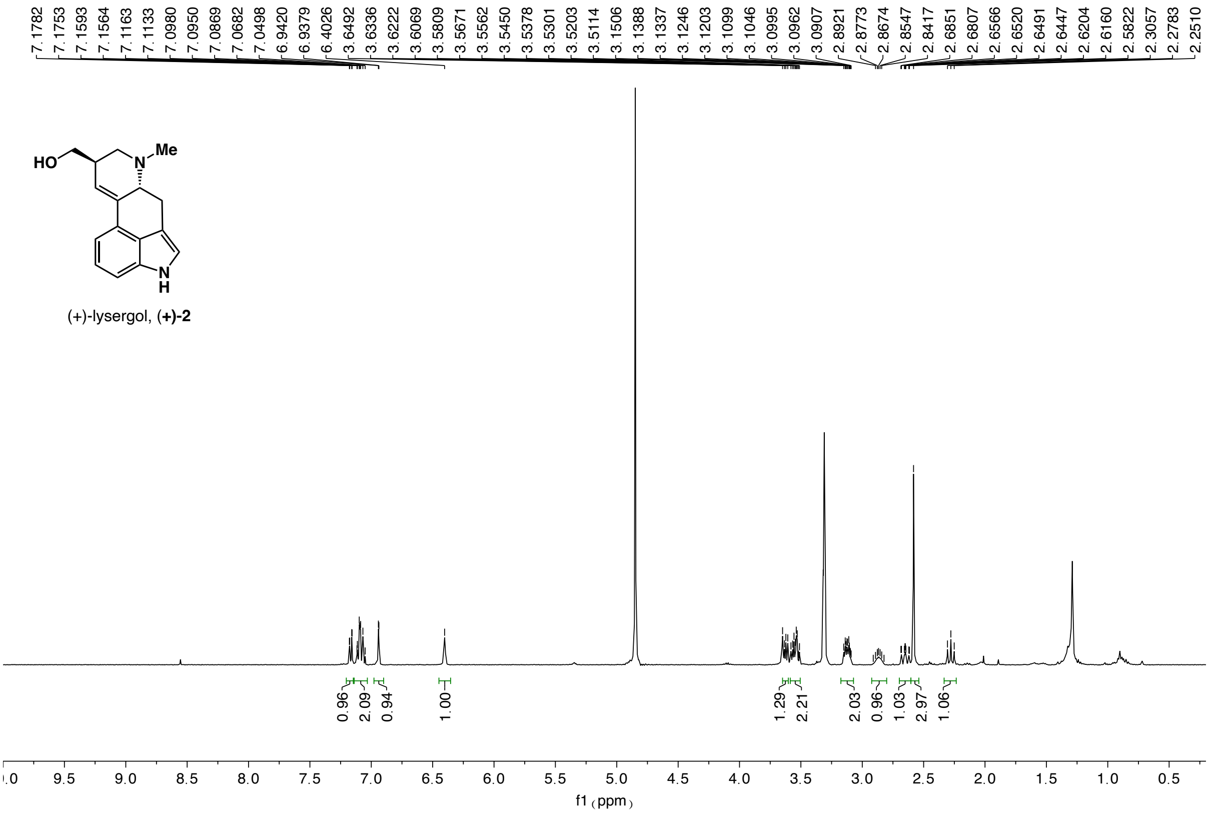

^{1}H NMR spectrum (400 MHz, MeOH-d_4) of compound (+)-2
